# Accounting for methodological variabilities in disease outbreak surveillance using wastewater-based epidemiology: a case study with SARS-CoV-2

**DOI:** 10.1007/s00216-026-06712-x

**Published:** 2026-08-08

**Authors:** James Boxall-Clasby, Nicola Ceolotto, Marjorie J. Gibbon, Kishore K. Jagadeesan, Edward J. Feil, Theresa Smith, Megan Robertson, Richard Standerwick, Ruth Barden, Julian Faraway, Barbara Kasprzyk-Hordern

**Affiliations:** 1 Department of Chemistry, University of Bath, Bath, BA2 7AY UK; 2 Centre of Excellence in Water-Based Early Warning Systems for Health Protection, University of Bath, Bath, BA2 7AY UK; 3 Institute for Sustainability, University of Bath, Bath, BA2 7AY UK; 4 Milner Centre for Evolution, Department of Life Sciences, University of Bath, Bath, BA2 7AY UK; 5 Centre for Mathematical Biology, University of Bath, Bath, BA2 7AY UK; 6 Department of Mathematical Sciences, University of Bath, Bath, BA2 7AY UK; 7 Wessex Water, Bath, BA2 7WW UK; 8 Environment Agency, Horizon House, Deanery Road, Bristol, UK

**Keywords:** SARS-CoV-2, RT-qPCR, Disease outbreak, Public health, Wastewater-based epidemiology, Analytical variability

## Abstract

Wastewater-based surveillance of SARS-CoV-2 has become an established approach for monitoring health status and disease outbreak at the community level. Although analytical protocols for RNA quantification have been widely implemented and optimised, the characterisation of variability introduced across all stages, such as storage, sample processing, and PCR quantification, remained limited, including its impact upon epidemiological interpretation. This study proposes an analytical framework to quantify variability across the complete workflow for SARS-CoV-2 RNA measurement in wastewater, including storage stability, sample processing, and PCR quantification. This framework was developed for a large SARS-CoV-2 dataset of two viral gene targets (N1, E-Sarbeco), which were quantified alongside human-associated CrAssphage for a total of 20,124 RT-qPCR data points derived from four water recycling centres (WRCs) over a 24-month period. This analysis revealed that sample refrigeration yielded richer epidemiological data than freezing, and that CrAssphage had minimal utility as a normalisation biomarker given its higher variability. Quantification of analytical variability for SARS-CoV-2 targets enabled development of a composite metric (Total Estimated SARS-CoV-2), improving sensitivity and reducing non-detects at the expense of reduced precision. This novel multi-stage framework had the capability to improve the sensitivity and interpretation of WBE data and the characterisation of WBE methodologies, with broad applicability for pathogen monitoring.

**Supplementary Information:**

The online version contains supplementary material available at https://doi.org/10.1007/s00216-026-06712-x.

## Introduction

The SARS-CoV-2 pandemic proved the urgent need to strengthen early-warning systems for disease outbreaks [1]. The lack of scalable community-level surveillance approaches at the start of the COVID-19 pandemic hindered effective tracking of disease spread [2], and demonstrated the necessity of surveillance systems capable of real-time reporting to support outbreak control at both a community and global scale [3].

Wastewater-based epidemiology has emerged as a valuable population-level surveillance approach in municipal wastewater, enabling early detection of infection trends, assessment of outbreak dynamics, and surveillance independent of clinical testing coverage, since wastewater is a pooled sample of analytical targets excreted by the population into the sewer network, and allows for a cheap and non-invasive assessment of community health [4]. As a result, wastewater monitoring has been incorporated into public health surveillance frameworks across countries for indicators of oxidative stress [5, 6], lifestyle factors [7, 8], illicit drugs [9, 10], and nucleic acids from infectious pathogens such as viral or bacterial DNA and RNA [5, 11, 12]. This approach enables the early detection of pathogens associated with disease cases that may be presymptomatic or asymptomatic at a community level [13, 14]. During the SARS-CoV-2 pandemic, WBE offered a non-invasive, near real-time, and cost-effective approach to monitor the prevalence of viral infections across communities, independently of individual testing or access to healthcare.

Detection of pathogenic RNA/DNA in wastewater involves multiple sequential stages, including sample storage, processing, and RT-qPCR analysis. Each stage introduces potential sources of variability, and although individual components of the analytical workflow have been extensively evaluated, such as viral recovery rates, limits of detection, and PCR inhibition, most investigations have focused on isolated steps rather than on integrated end-to-end variability. The cumulative nature of methodological variability has important implications for epidemiological interpretation. Wastewater viral concentrations are frequently interpreted as proxies for changes in community infection prevalence. Without a clear understanding of methodological uncertainty, it becomes difficult to distinguish whether observed differences in concentrations reflect actual changes in population-level infection dynamics or simply artefacts introduced across analytical workflows. For example, there might be differences in assay sensitivity, specificity, and inhibition resistance across varying methodological parameters such as reagent selection, primer design, and RT-qPCR settings [15]. Multiple papers have been published discussing these challenges and report three key strategies for managing this variation: (i) the use of replicates, (ii) the use of controls, and (iii) the appropriate interpretation of non-detects [14–18]. These factors could significantly influence the accuracy and reliability of biological measurements in wastewater, highlighting the need to systematically assess and manage variability across all steps of the workflow. Bridging this gap is essential to enable full method characterisation, ensuring the interpretability of WBE data for disease outbreak tracking; therefore, a comprehensive assessment of multi-stage variability is necessary to support wastewater surveillance.

**Fig. 1:**
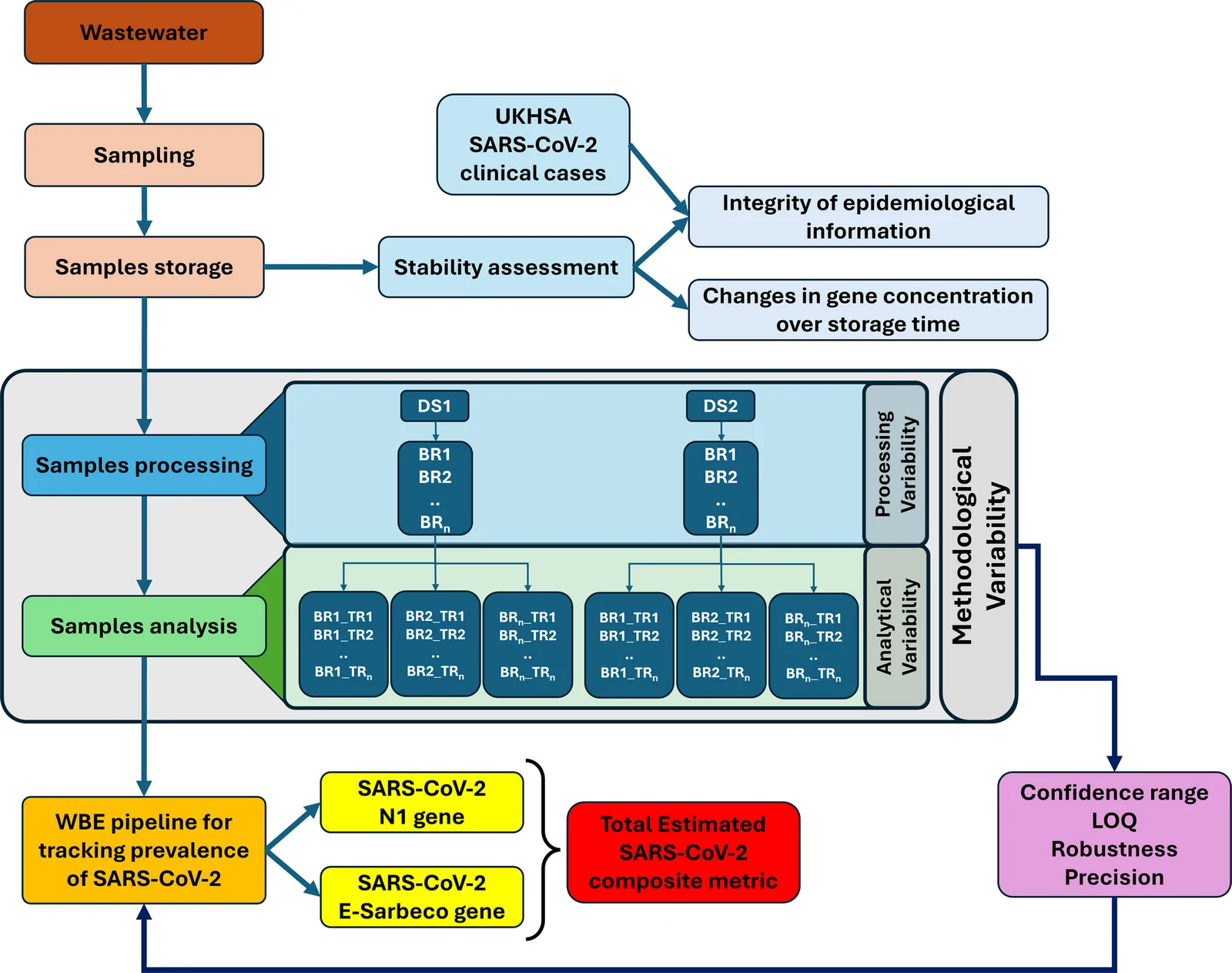
Novel analytical framework to characterise method variability in quantification of SARS-CoV-2 N1, E-Sarbeco, and CrAssphage using RT-qPCR. This workflow included stability assessment to identify optimal storage conditions to preserve epidemiological information and methodological variability of wastewater samples (DS, daily sample; BR, biological replicate; TR, technical replicate)

The objective of this study was to develop a novel analytical framework (Fig. 1) to characterise method performance of RT-qPCR quantification in influent wastewater samples. Two SARS-CoV-2 gene targets were used, N1 and E-Sarbeco, and CrAssphage was included as a trial biomarker for population normalisation. The following methodological steps were assessed:

- Storage stability: stability of gene targets was assessed (a) at +4 °C (refrigeration) for seven time points covering 4 weeks, (b) at −20 °C (frozen) for ten time points covering 1 year, (c) following up to four freeze/thawing (FT) cycles.
- Integrity of epidemiological information under methodological conditions: comparing SARS-CoV-2 prevalence estimated via WBE with clinical case data for COVID-19 incidence to evaluate whether storage conditions changed gene target detectability in wastewater and thereby affected early-warning effectiveness.
- Methodological variability: evaluating variability across the analytical workflow (from sample preparation to RT-qPCR analysis) to identify sources of uncertainty, establishing error bars and determining the limit of quantification (LOQ) for SARS-CoV-2 gene targets in wastewater.
- Composite metric for disease prevalence: development of a composite metric (Total Estimated SARS-CoV-2) comprising data from the two gene targets to determine whether it could model COVID-19 prevalence more effectively than the SARS-CoV-2 N1 and E-Sarbeco gene targets used individually.

## Materials and methods

### Samples collection

**Fig. 2:**
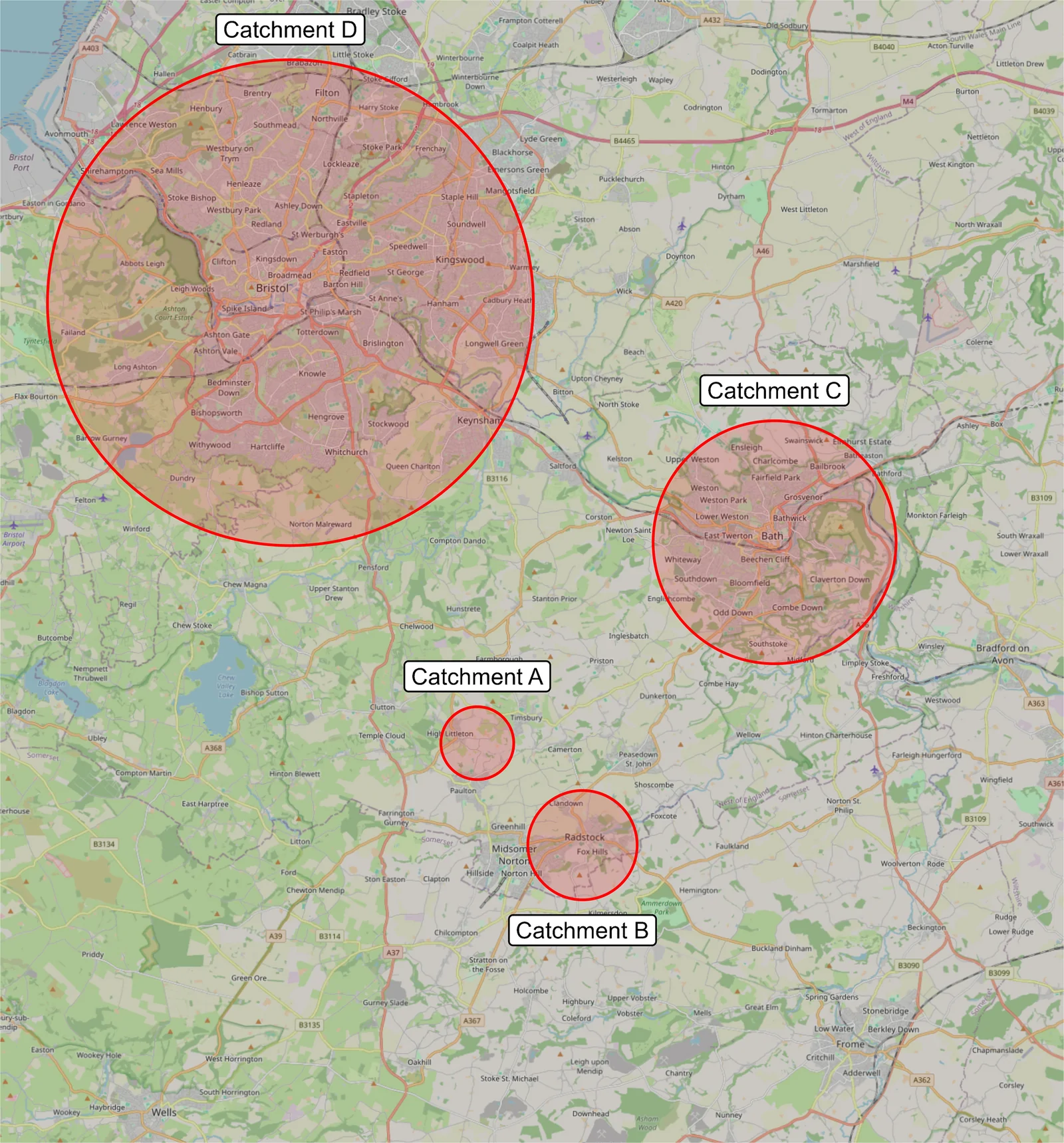
WRCs catchment areas included in this study: Paulton (catchment A), Radstock (catchment B), Bath (catchment C), and Bristol (catchment D)

Influent wastewater was collected in polyethylene terephthalate plastic bottles between April 2020 and February 2022 from four wastewater recycling centres (WRCs) in the South West of England, corresponding to the population centres of Paulton (A), Radstock (B), Bath (C), and Bristol (D), for a total of 797 daily samples (DS). A map of the wastewater catchment regions is reported in Fig. 2.

Samples were collected using a randomised sample collection approach (two samples per week) via 24-h composite sampling using Teledyne ISCO 5800 and Teledyne ISCO 6712. Sub-samples were collected once every 15 min (WRC A, B, and C) or 60 min (WRC D) for 24 h, with either time-proportional (WRC A, B, and D) or flow-proportional sampling (WRC C) regimes. After 24 h, all sub-samples were pooled and mixed for subsequent analysis. The difference between sampling regimes at different WRCs was due to the differing availability of autosamplers at the different WRCs studied. Samples were refrigerated during transportation and then stored until sample processing, typically at +4 °C or −20 °C.

### Reagents and equipment

Centrifugation for sample preparation was carried out using an Eppendorf 5910R centrifuge with a swinging bucket rotor, 50-mL conical adaptors and 50-mL sterile conical centrifuge tubes. Sterile polypropylene 50-mL conical centrifuge tubes (Greiner) were purchased from Merck. Centrifugation for nucleic acid extraction was carried out using Thermo Scientific Pico 17 Microcentrifuge with 1.5-mL RNase-free microfuge tubes. RNase-free microfuge tubes (Invitrogen) were purchased from Thermo Fisher Scientific. Sample mixing was carried out using the SLS Lab Basics Vortex Mixer. Nucleic acid extraction was carried out using the New England Biolabs™ Monarch RNA Clean-up Kit (50 µg) (New England Biolabs™ T2040), following the New England Biolabs™ Supplemental Protocol (Purification of RNA from the Aqueous Phase Following TRIzol/Chloroform Extraction). Isopropyl alcohol, phosphate-buffered saline tablets, PEG-8000, and PBS pellets were purchased from Merck. TRIzol™ (Invitrogen) and chloroform were purchased from Thermo Fisher Scientific. A synthetic 519 nucleotide (nt) DNA fragment harbouring all four target sequences was purchased as a custom GeneStrand from Eurofins. Applied Biosystems™ MicroAmp™ Fast Optical 96-well reaction plates (0.1 mL) were purchased from Thermo Fisher Scientific. Adhesive optical Microseal ‘B’ PCR plate Sealing Film was obtained from Biorad. RT-qPCR analysis was carried out using an Agilent AriaMX Real-Time PCR system. Heat-inactivated SARS-CoV-2 was deposited by the US Centers for Disease Control and Prevention (US CDC) and obtained from LGC through BEI Resource, NIAD, NIH: Genomic RNA from SARS-Related Coronavirus 2, Isolate USA-WA1/2020, NR-52285.

### Samples processing

Samples were decanted as three 40 mL biological replicates in 50-mL centrifuge tubes. Each replicate was pasteurised over 90 min in a water bath at 60 °C to reduce potential sample infectivity [19]. Suspended particulate matter was then removed by centrifugation at 4500 *g* for 30 min at 4 °C, and the supernatant was retained. Concentration was carried out using a modified version of the PEG-centrifugation methodology previously described by Wu et al. [19]. A PEG/PBS stock solution was prepared by dissolving 80 g of PEG-8000 (0.4 g/mL) in 200 mL of phosphate-buffered saline (PBS, 5×). Ten milliliters of PEG/PBS solution was added to each 40 mL replicate of supernatant (creating a solution of PEG-8000 80 mg/mL and PBS 1×), whereupon each replicate was incubated overnight at 4 °C on a roller mixer to allow precipitation. After centrifugation (20,000 *g* for 60 min at 4 °C), the supernatant was discarded and the pellet was dissolved in 1 mL of TRIzol™ reagent and transferred to a 1.5-mL microcentrifuge tube. These samples were stored at −80 °C until nucleic acid extraction.

### Nucleic acid extraction

Nucleic acid extraction was carried out using TRIzol™. Each 1 mL replicate in TRIzol™ was thawed and incubated at room temperature for 5 min, whereupon 200 μL of chloroform was added and each replicate was shaken and incubated at room temperature for 3 min. Each replicate was then centrifuged for 15 min at 12,000 *g* at 4 °C and the upper aqueous phase was retained and transferred to a new microcentrifuge tube. Each nucleic acid replicate then underwent spin column-based purification using the Monarch® RNA Clean up Kit following the New England Biolabs™ Supplemental Protocol (Purification of RNA from the Aqueous Phase Following TRIzol/Chloroform Extraction). According to the manufacturer’s protocol, 500 μL of RNA clean-up wash buffer was then added to the clean-up column, which was then centrifuged for 1 min at 16,000 *g* at room temperature whereupon the flow-through was again discarded. To ensure complete removal, residual wash buffer was removed by an additional 1-min centrifugation at 16,000 *g* at room temperature. Each replicate of purified nucleic acid was eluted in 50 μL of nuclease-free water and stored at −80 °C until analysis was carried out.

### Nucleic acid quantification by RT-qPCR

Biological replicates of purified nucleic acid were analysed as individual technical replicates by multiplex RT-qPCR analysis using an Agilent AriaMX Real-Time PCR system. Enzymes (GoScript Enzyme Mix) and custom master mix (GoTaq Wastewater Master Mix). SARS-CoV-2 RNA was quantified via two gene targets: the nucleocapsid gene using the US CDC 2019-nCoV_N1 (N1) primers and probe, and a region of the envelope gene using the Charité E-Sarbeco (E-Sarbeco) primers and probe. A single target was used to identify CrAssphage (Table 1). The dyes and quenchers from these assays were adapted to allow multiplexing, shown in Table 1. The RT-qPCR SARS-CoV-2 sequences are detailed alongside the SARS-CoV-2 genome in Supplementary Information. RT-qPCR analysis was conducted using 96-well plates, sealed using optically clear adhesive plate covers. Each PCR well contained 2.5 µL of sample, 5 µL of GoTaq Wastewater Probe qPCR Master Mix (custom), 0.2 µL of GoScript Enzyme Mix, and primers/probes at concentrations described in Table 1. The reaction volume was adjusted to 10 µL with nuclease-free water. Thermal cycling reactions were carried out as recommended for the GoTaq Wastewater Probe 1-step RT-qPCR system: 15 min at 45 °C, 2 min at 95 °C followed by 50 cycles of 95 °C for 3 s and 60 °C for 30 s. Data were collected at end of each 60 °C annealing/extension step. Because SARS‑CoV‑2 is an RNA virus, whereas CrAssphage is a DNA bacteriophage, only the SARS‑CoV‑2 targets require a reverse-transcription step. CrAssphage can be quantified directly by qPCR, but its inclusion within the RT-qPCR workflow does not impede amplification and therefore allows both targets to be processed in parallel. Each 96-well plate included triplicate calibrators and controls. Calibration was carried out using serial dilutions of a synthetic 519 nucleotide DNA fragment harbouring the target sequences, ranging from 1.0 to 1.0^5^ gene copies per µL (gc/µL). Two dilutions of non-extracted heat-inactivated SARS-CoV-2 were used as qPCR positive controls. No-template controls (NTCs) of nuclease-free water were used as qPCR negative controls. Measurements below 1.0 gc/µL were classified as non-detects. Plates were removed from subsequent analysis when either (i) at least two of the three NTCs resulted positive, with a concentration ≥ 1 gc/µL or a C_T_ < 31 or (ii) at least two of the three positive controls yielded non-detect measurements.

**Table 1:** RT-qPCR gene targets with assay names and origins with corresponding primer/probe names, sequences, and concentrations

RT-qPCR target	Source, reference, and alteration from source	Name	Sequence	Concentration in well (nM)
SARS-CoV-2 nucleocapsid gene (SARS-CoV-2 N1)	US CDC 2019-nCoV_N1 (unaltered) [20]	2019-nCoV_N1-F	GACCCCAAAATCAGCGAAAT	250
		2019-nCoV_N1-R	TCTGGTTACTGC CAGTTGAATCTG	250
		2019-nCoV_N1-P	6FAM-ACCCCGC ATTACGTTTGGT GGACC-BHQ1	62.5
SARS-CoV-2 envelope gene (SARS-CoV-2 E-Sarbeco)	Charité E-Sarbeco (changed dye and quencher) [21]	E-Sarbeco-F	ACAGGTACGTTA ATAGTTAATAGC GT	200
		E-Sarbeco-R	ATATTGCAGCAG TACGCACACA	200
		E-Sarbeco-P1	HEX-ACACTAGC CATCCTTACTGC GCTTCG-BHQ1	100
crAssphage	Stachler et al. 2017 (changed dye and quencher) [22]	056F1	CAGAAGTACAAACTCCTAAAAAACGTAGAG	450
		056R1	GATGACCAATAAACAAGCCATTAGC	450
		056P1	Cy5- AATAACGATTTACGTGATGTAAC-BHQ3	40

The target gene concentration for each technical replicate (*TR*_*g*c_, gc L^−1^) was calculated using Eq. 1, where *WC*_*gc*_ (gc µL^−1^) is the well concentration for the target gene and *f* is the concentration factor (µL L^−1^).

1 $${TR}_{gc}(\mathrm{g}\mathrm{c} {\mathrm{L}}^{-1})={WC}_{gc}\bullet f$$

### Data processing

#### Gene target quantification

The mean target gene concentration was determined for each biological replicate (*BR*_*gc*_, gc L^−1^) by the geometric mean of its *TR*_*gc*_ using Eq. 2:

2 $${BR}_{gc}(\mathrm{g}\mathrm{c} {\mathrm{L}}^{-1})=\sqrt[n]{{TR}_{gc1}\times {TR}_{gc2}\times \dots {TR}_{gcn}}$$

The geometric mean was used as it is appropriate for datasets characterised by high variability and spanning multiple orders of magnitude (such as qPCR, an inherently multiplicative methodology), which is mathematically analogous to the use of the geometric mean of qPCR cycle threshold (C_T_). Similarly, target gene concentration for each daily wastewater sample (*DS*_*gc*_, gc L^−1^) was estimated through the geometric mean of its component biological replicate concentrations, *BR*_*gc*_ using Eq. 3:

3 $${DS}_{gc}(\mathrm{g}\mathrm{c} {\mathrm{L}}^{-1})=\sqrt[m]{{BR}_{gc1}\times {BR}_{gc2}\times \dots {BR}_{gcm}}$$

In the calculation of *BR*_*gc*_ and *DS*_*gc*_, non-detect measurements were removed. If all technical replicates for a biological replicate were non-detects, then that biological replicate was also considered a non-detect. Similarly, if all biological replicates for a given sample were non-detects, then that daily sample was also considered a non-detect.

The removal of non-detect replicates constitutes a *complete case* approach and may introduce bias in low concentration contexts. Indeed, when the true target concentration is low, only a small subset of TRs for a given BR may exceed the limit of detection, and only those detected values are taken forward for the calculation of BR and DS. Excluding non-detects in this way may thus inflate the estimated concentration of the target. Multiple replicates may mitigate this effect to an extent, but they cannot eliminate it entirely. Although there are these limitations, this approach was adopted as a practical compromise for simplicity and consistency.

#### Assessment of stability and epidemiological integrity

The impact of storage of influent wastewater on quantification of the SARS-CoV-2 N1 and E-Sarbeco targets was assessed in triplicate as described in sections ‘Sample processing’ and ‘Nucleic acid extraction’. The impact on SARS-CoV-2 recovery was assessed under the following conditions:

- The default case was immediate processing without storage (day 0 with 0 freeze/thaw cycles).
- Refrigerated stability was assessed upon storage at +  4 °C with six time points (days 1, 3, 7, 14, 21, and 28)
- Frozen stability was assessed upon storage at − 20 °C with nine time points (day 3 and weeks 1, 2, 4, 8, 13, 26, 39, and 52)
- Freeze/thawing stability was assessed over four FT-cycles, wherein each FT-cycle comprised placing the sample in the freezer for ≥ 3 days prior to thawing at room temperature.

A complementary analysis was also undertaken to evaluate the epidemiological informativeness of wastewater-derived data obtained via different storage regimes within a surveillance campaign. This was conducted by comparing the *per capita* COVID-19 case incidence (UKHSA clinical data) against the corresponding concentrations of the SARS-CoV-2 N1 and E-Sarbeco gene targets (gc/L) in wastewater obtained via different storage conditions (refrigerated, frozen, freeze-thaw cycled). The sensitivity of the WBE method was assessed for the different sample storage approaches, with a selected cut-off for COVID-19 case detection of 250 COVID-19 cases per million people (cpm) within the WRC catchment, as derived from UKHSA data. The resulting scatterplot was split into a 2 × 2 matrix, with the concentrations split to give 95% specificity for an incidence per capita of 250 cpm: a high specificity was selected for the minimisation of false alarms for incipient disease outbreak detection. Within this confusion matrix, the sensitivity of the WBE method may be determined for the detection of COVID-19 prevalence exceeding 250 CPM.

#### Characterisation of method variability

Variability was observed for native gene targets by analysing replicates across a large dataset. The continuous variability, describing the spread of measured concentrations among ‘detect’ replicates was assessed by analysing the distribution of replicate concentration ratios:

- Processing variability (PV) was measured by the variability in the ratio of each daily sample to its component biological replicates (DS:BR).
- Analytical variability (AV) was measured by the variability in the ratio of each biological replicate to its component technical replicates (BR:TR).
- Methodological variability (MV) was measured by the variability in the ratio of each daily sample to its component technical replicates (DS:TR).

To avoid underestimation of variability, BRs with only a single TR component ‘detect’ were removed from this calculation as they could not contribute any information about variability.

Beyond *continuous variability*, *discrete variability* was also characterised, describing the probability of a target-specific non-detect for a replicate, determined by analysing the distribution of ‘non-detects’ across the concentration range. Both continuous and discrete variability characterisation approaches were summarised in Table 2.

**Table 2:** Summary of the approach used for characterising variability across different methodological stages. Both continuous variability and discrete variability were assessed BR (biological replicates), TR (technical replicates), and DS (daily sample)

	Analytical variability (AV) Portion of variability related to RT-qPCR analysis	Processing variability (PV) Portion of variability related to sample processing	Methodological variability (MV) Complete variability inherent to the method, comprising both AV and PV	Observed as	Plotted as
Continuous variability	Variability in BR:TR ratios for a target	Variability in DS:BR ratios for a target	Variability in DS:TR ratios for a target	The range of measured concentrations between ‘detect’ replicates for a given sample and target	(a) Scatterplots (distribution of ratios on a logarithmic scale vs concentration range) (b) Histograms (log-transformed ratios vs frequency)
Discrete variability	Proportion of TR non-detects within a detect BR for a target	Proportion of BR non-detects within a detect DS for a target	Proportion of TR non-detects within a detect DS for a target	Proportion of ‘non-detect’ replicates, determined per concentration-range	Bar charts (proportion of non-detect replicates vs concentration range)

From scatterplots of these ratios within *continuous variability* analysis, the mean value (*μ*, equal to 1) was visualised alongside the upper confidence limit (UCL, *µ* + 2*σ*) and lower confidence limit (LCL, *µ* – 2*σ*). These values were used to calculate the confidence range (CR), linear-adjusted standard deviation (*σ*_linear_), and coefficient of variation (%CV) as from Eqs. 4 to 6:

4 $$CR= \frac{UCL}{\mu }= \frac{\mu }{LCL} \left(\mu =1\right)$$

5 $${\sigma}_{\mathrm{l}\mathrm{i}\mathrm{n}\mathrm{e}\mathrm{a}\mathrm{r}}= \frac{UCL-LCL}{4}$$

6 $$\%CV= \frac{{\sigma}_{\mathrm{l}\mathrm{i}\mathrm{n}\mathrm{e}\mathrm{a}\mathrm{r}}}{\mu } \bullet 100 (\mu =1)$$

Conventionally, limit of detection (LOD) is defined using the variability of a blank sample. In PCR, the calibrated range cannot include a concentration of zero because it is performed on a logarithmic scale; consequently, blank samples are expected to yield non-detects, and a standard deviation could not be estimated for these measurements. The LOD for extracted nucleic acid (LOD_gc/µL_) is defined as the bottom calibrator (1 gc/µL), which is concentration below which measurements are categorised as non-detects: this provides an internal coherence in the treatment of non-detects.

Limit of quantification (LOQ_gc/L_) is usually defined by using the variability of a spiked control. However, in this work an alternative approach was proposed using the variability of native targets within the wastewater matrix, allowing the method to be characterised under real conditions.

Aligning with 2002/657/EC and ISO-11843 [23] protocols, the LOQ corresponds to the 95% one-sided confidence limit above the limit of detection (LOD), or LOD + 1.64σ assuming additive variability, which is the case for log-transformed data. Conversely, for untransformed RT-qPCR data, variability is multiplicative and concentration-dependent: as such we may estimate variability using the 95th percentile of the methodological variability (DS:TR) ratio distribution (MV_95%_) to obtain the LOQ value for extracted nucleic acid (LOQ_gc/µL_) using Eq. 7, where *LOD*_gc/µL_ is 1 gc/µL, making the LOQ effectively a function of MV_95%_. LOQ values for wastewater samples (LOQ_gc/L_) are then calculated using Eq. 8, with the concentration factor (CF) of 1,250 µL/L applied to convert between units.

7 $$LOQ_{gc/\mu L} = LOQ_{gc/\mu L} \bullet \frac{MV_{95}}{MV_{50}}$$

8 $${LOQ}_{\mathrm{g}\mathrm{c}/\mathrm{L}}= {LOQ}_{\mathrm{g}\mathrm{c}/\upmu \mathrm{L}} \bullet CF$$

With this approach, no more than 5% of technical replicate measurements for non-detect DSs are expected to exceed the LOQ, while no more than 5% of TR measurements for daily samples above the LOQ are anticipated to yield non-detects. Based on methodological variability CR values, upper and lower confidence limits were calculated for DS measurements (UCL and LCL, respectively) per Eqs. 9–10, where *C*_gc/L_ is the DS concentration.

9 $$UCL= {C}_{\mathrm{g}\mathrm{c}/\mathrm{L}}\bullet {CR}_{MV}$$

10 $$LCL= \frac{{C}_{\mathrm{g}\mathrm{c}/\mathrm{L}}}{{CR}_{MV}}$$

### Composite metric for disease prevalence

The composite metric Total Estimated SARS-CoV-2 was developed to provide a multivariate model for a more rounded assessment of SARS-CoV-2 prevalence. The Total Estimated SARS-CoV-2 concentration in a BR was estimated by first taking the non-detect-removed geometric mean of TR concentrations between all SARS-CoV-2 gene targets (i.e. N1 and E-Sarbeco), with each measurement weighted in inverse proportion to the %CV for the respective gene target. The Total Estimated SARS-CoV-2 concentration for the DS was then calculated by the non-detect-removed geometric mean of the component BR concentrations for this composite target.

13 $${BR}_{gc}(\mathrm{g}\mathrm{c} {\mathrm{L}}^{-1})=\sqrt[\sum {w}_{i}]{{\left({TR}_{gc1}\right)}^{{w}_{1}}\times {\left({TR}_{gc2}\right)}^{{w}_{2}}\times \dots {\left({TR}_{gcn}\right)}^{{w}_{n}}}$$

A weighting function inversely proportional to %CV was used to obtain a consistent weighting across the calibrated range, as relative variance was consistent in proportion to the measured concentration: the alternative use of the absolute variance (1/*σ*^2^) was not viable, resulting in an over-valuation of lower values. Using 1/(%CV)^2^ = *μ*^2^/*σ*^2^ made the weighting function agnostic to the order of magnitude of the measured sample concentration, preventing bias towards lower concentrations, while giving greater weight to less variable targets as intended.

## Results

### Impact of storage condition upon gene targets quantitation

The stability of refrigerated influent wastewater samples was assessed at five time points (day 0, day 7, day 14, day 21, day 28), as it was determined that samples would not be retained longer than this in the refrigerator prior to processing. Indeed, in Figure S1(a-c), (i) no overall trend was observed in stability, (ii) the average standard deviation followed a decreasing order (CrAssphage > E-Sarbeco > N1), and (iii) non-detects did not affect most measurements. These results align well with other studies [24–26], as well as with identified good practice during the COVID-19 pandemic, and US NWSS 2024 guidelines [27], which recommended immediate sample processing, and if necessary to store samples at + 4 °C for up to 4 days [27–29]. Thus, storage of wastewater for subsequent processing for RT-qPCR analysis might be considered a viable solution and feasible for low-resource environments, avoiding any requirement to freeze samples.

The stability of frozen wastewater samples was assessed over a year (ten time points: day 0, day 1, 1 week, 2 weeks, 4 weeks, 8 weeks, 13 weeks, 26 weeks, 39 weeks, and 1 year) to consider the impact of longer-term storage upon late-processed and archived samples, common practices in WBE. Numerous non-detects and high variability were observed both within and between timepoints, with an absence of clear stability trends. This effect was particularly evident for E-Sarbeco and CrAssphage (observed in Figure S2(a-c)), as biological replicates for these targets showed higher prevalence of non-detects with few detected measurements, independent of the time spent frozen. Nevertheless, the variability at − 20 °C for all targets was greater than that observed following storage at 4 °C. Average standard deviation was higher for CrAssphage, followed by E-Sarbeco and N1. Since this behaviour was observed across all time points, it could be implied that it was not time in the freezer that caused degradation within individual biological replicates, but rather the freeze/thaw process itself.

On the basis of this stability study, wastewater sample storage at − 20 °C could not be considered the optimal choice due to the presumed deleterious effect on target recovery. This finding was in line with other studies [26, 28, 30] and with the detailed 2024 stability study from Williams et al., who found that SARS-CoV-2 N, E, and S genes degraded in wastewater upon freeze/thaw-cycling, showing reduced RNA integrity compared to samples stored at 4 °C [31]; on the other hand, these findings showed opposite results to Hokajarvi et al. which indicated that N2 and E-Sarbeco SARS-CoV-2 targets were stable in wastewater stored at − 20 °C for 58 days [32]. In addition, the stability of wastewater samples was complemented with up to four FT-cycles to further elucidate the stability under frozen condition. The data presented in Figure S3a shows that repeated freeze/thawing substantially reduces SARS-CoV-2 E-Sarbeco recovery which has a median result of a non-detect for all FT stability samples that had undergone at least 1 freeze/thaw sample. Conversely, the SARS-CoV-2 N1 target appeared more resilient to FT cycling in Figure S3b, despite the intra- and inter-timepoint variability observed, aligning with Fig. S2b and previous study by William et al. [31] and Thapar et al. [30]. The median value observed for CrAssphage upon freeze/thaw cycling never exceeds FT = 0 value, which may indicate FT instability. Therefore, FT cycling demonstrably had a negative impact on target stability and should be avoided if possible.

Since refrigerated conditions exhibited a reduced range of variability and lower occurrence of non-detects compared to frozen conditions, refrigeration was recommended as the optimal choice for RNA storage up to seven days for public health monitoring purposes. This recommendation was based on the observed absence of non-detects and lower variability, which was interpreted as an indicator of reliable and potentially safer storage within the conditions tested in this study. However, it was important to note that stability outcomes did not offer any evidence regarding the broader implications of storage conditions on surveillance and its potential consequences for public health. Specifically, this analysis did not account for long-term monitoring, intra- and inter-catchments variability (flow rates and population size) that may underline further implications which are discussed in the next section.

### Epidemiological integrity assessment upon storage conditions

Although refrigeration seemed to be preferable to frozen storage as discussed in the previous section, the actual impact of sample storage conditions upon the generation of reliable epidemiological data for public health insights (e.g. early disease detection, assessment of surveillance infrastructures, and identification of policy impacts) required further investigation.

In particular, WBE targets a wide range of pathogens and genetic markers, and it is essential to understand how storage conditions influence the epidemiological interpretability of wastewater measurements and therefore impact the disease outbreak tracking across different targets. To address this, an epidemiological integrity assessment was conducted by comparing prevalence of SARS-CoV-2 in wastewater with the corresponding UKHSA-derived COVID-19 clinical cases for the WRC catchments on the day of sampling. Beyond evaluating SARS-CoV-2 specifically, this analysis aimed to assess how storage conditions may influence the epidemiological performance of wastewater surveillance more broadly, providing insights and an additional control applicable to other pathogen targets within WBE programmes.

**Fig. 3:**
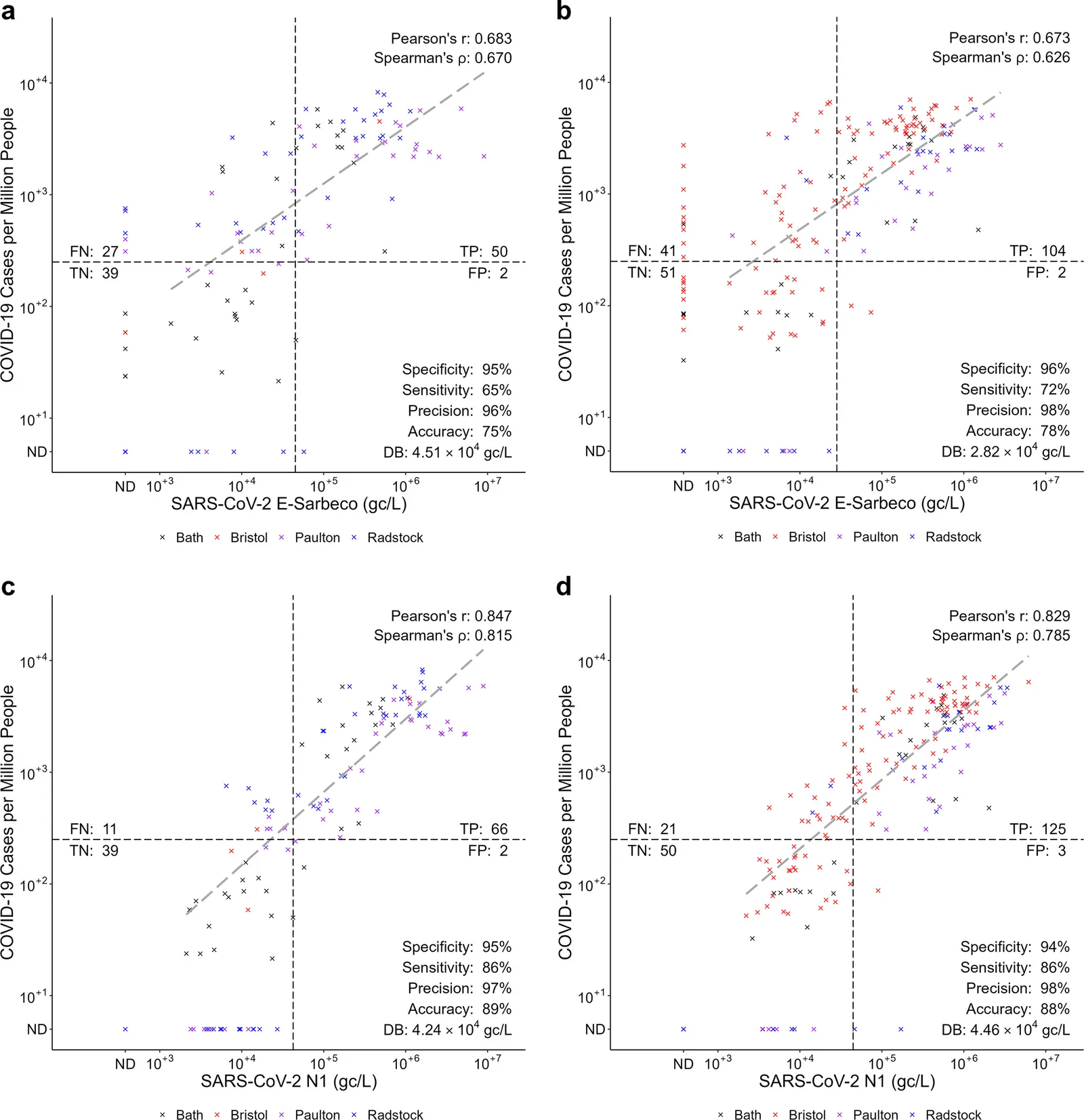
**a**–**d** Scatter plot of SARS-CoV-2 E-Sarbeco (**a**, **b**) and N1 targets (**c**, **d**) at 4 °C (FT = 0) in wastewater samples for 0–1 day and 2+ days. *C*_*gc*_ (gc/L) is plotted against COVID-19 cpm for that day. Data points are grouped by WRC. In the top and bottom right corners, the performances of the model are reported

Wastewater sample stability at 4 °C was analysed and sensitivity was determined as described in Data processing section. The sensitivity data are provided graphically in Fig. 3a–d below. Under refrigerated storage, the concentration of SARS-CoV-2 N1 and E-Sarbeco targets in wastewater exhibited a linear relationship with UKHSA-derived COVID-19 caseload on the day of samples collection, with Pearson’s *r*-values of 0.67 and 0.83 for SARS-CoV-2 E-Sarbeco and N1, respectively, increasing to 0.68 and 0.84 when stored no longer than 1 day before processing. Negligible divergence was observed among sites. This linearity indicated good discriminatory capabilities across the measured concentration range; moreover, the WBE methodology exhibited consistent 86% and 74–77% sensitivities for elevated COVID-19 case detection for N1 and E-Sarbeco, respectively, for all durations at 4 °C, using a specificity threshold of 95%. The lower sensitivity for E-Sarbeco aligned with its higher variability in the traditional stability study, so this may be related to the intrinsic characteristics of the gene target itself. Further, the data was well aligned between the four studied catchments indicating that the observed stability of both N1 and E-Sarbeco might reasonably be considered representative of that expected from samples originating from different WRC catchments. Moreover, these data did not indicate the existence of any significant stability issues for wastewater stored at 4 °C for 2+ days, as compared to 0–1 days, since differences remained within acceptable limits and retained sufficient discriminatory power and reliability. These results suggested that the method demonstrated increased accuracy due to a reduced false-omission rate. At the same time, the number of false negatives indicated room for improvement in sensitivity. This improved sensitivity with consistent specificity is critical, particularly in surveillance for low-incidence infectious disease outbreaks: in such an application, even a highly specific methodology may result in a much higher number of false positives than true positives due to the relative rarity of the monitored disease. Overall, the confusion matrix provided a nuanced picture of performance, supporting the method’s general reliability while highlighting the need to optimise sensitivity while safeguarding a high level of specificity. This approach demonstrates the importance of target-specific performance considerations when extending WBE methodologies to additional pathogen targets.

**Fig. 4:**
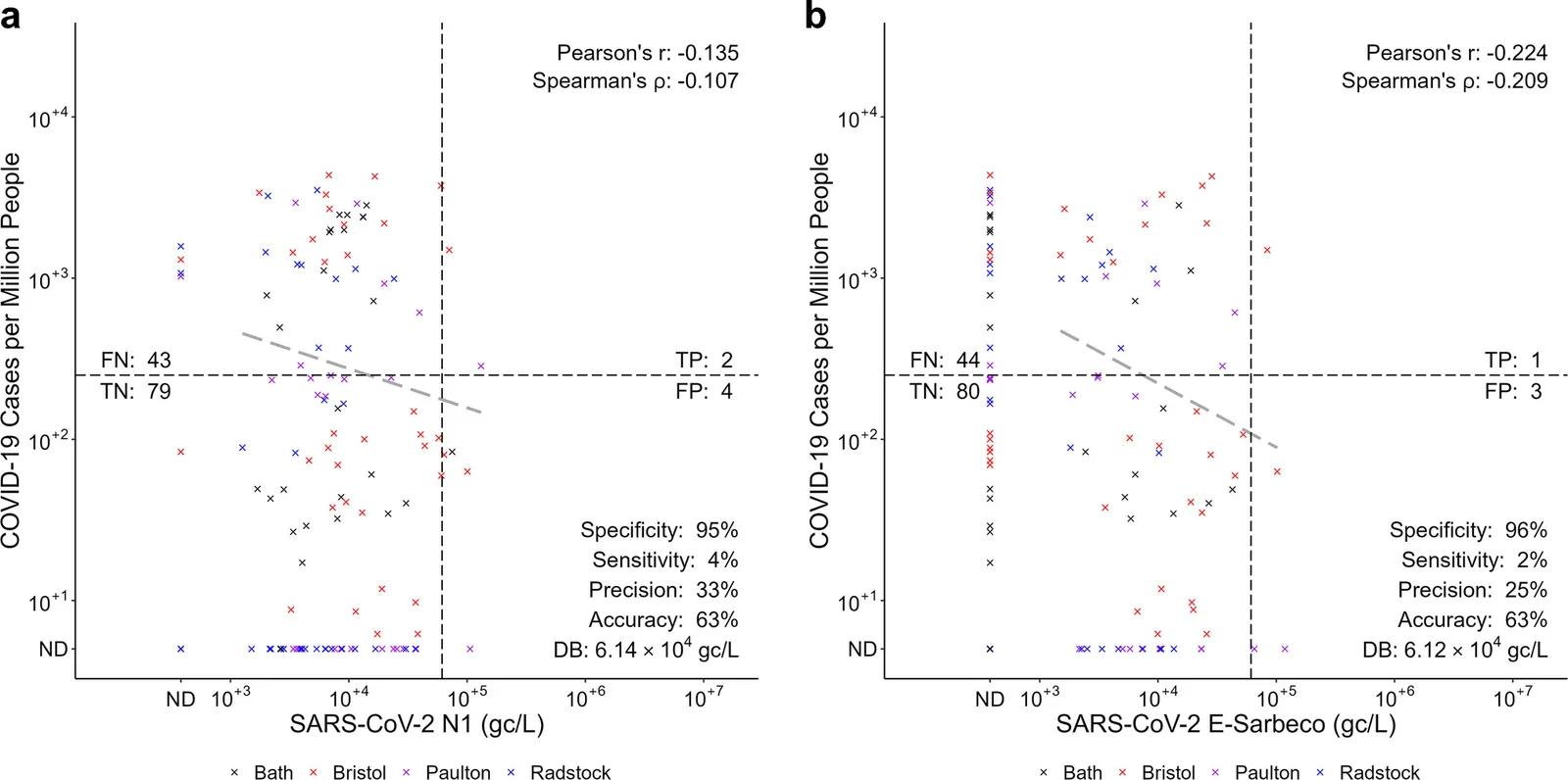
**a**, **b** Scatter plot of SARS-CoV-2 N1 and E-Sarbeco recovered from frozen (FT = 1) wastewater samples plotted against COVID-19 cpm for that same day. Data points are grouped by WRC. In the top and bottom right corners, the performances of the model are reported

Conversely, samples stored at − 20 °C exhibited significantly reduced sensitivity and quantification of both SARS-CoV-2 N1 and E-Sarbeco: within the plots in Fig. 4a, b, this was observed in the clustering of measurements on the left side of the plots. Thus, the quantified gene target from a frozen sample may be expected to fall within a wide concentration range, providing no epidemiological information about the caseload from that day. As the actual caseload could not be distinguished from gene concentration alone, sample freezing eradicates the capacity to use SARS-CoV-2 quantitation to assess prevalence at community level and provide meaningful epidemiological insights (e.g. early detection of viral outbreaks). Moreover, a significant drop in method performance was observed when comparing refrigerated and frozen storage for both SARS-CoV-2 N1 and E-Sarbeco, with precision (the proportion of positive results that were not false positives) falling from 98 to 33% for N1. Based on these observations, storage under frozen conditions has a clear deleterious impact upon the quantification of SARS-CoV-2 RNA; these findings agreed with section ‘Impact of storage condition upon analyte quantitation’ and with the stability study conducted by Williams et al., who found that genetic material in wastewater samples from the N, E, and S genes of SARS-CoV-2 degraded upon freeze/thaw-cycling, showing reduced RNA integrity compared to samples stored at 4 °C [33]. Therefore, frozen samples within this dataset provided minimal information about the COVID-19 case rate. Although the study conducted by Hokajarvi et al. indicated that N2 and E-Sarbeco SARS-CoV-2 targets were stable in wastewater stored at − 20 °C for 58 days [32], it must be acknowledged that 86% of data included in the epidemiological integrity assessment described in this section were stored at − 20 °C for more than 58 days, with an average storage time of 268 days. The impact of storage time at − 20 °C for these (FT = 1) samples could not be determined since no correlation between gene quantitation and caseload could be identified for these frozen samples, regardless of storage time.

### Characterisation of variability

Typically, the variability for a target within a methodology might be assessed through analysing the performance of non-native process controls [34, 35]. The size of this dataset and the breadth of replicate data enabled an alternative approach, however, whereby the variability was assessed for *native* gene targets. The approach used for the characterisation of variability within this manuscript was similar to that utilised by Wade et al. for the visualisation of SARS-CoV-2 N1 gene variability between replicates [36], albeit with three innovations: (i) the *x*-axis used the concentration instead of the C_T_ value, (ii) the *y*-axis displayed the multiplicative difference between concentrations instead of the (analogous) additive difference between C_T_ values, and (iii) the difference was here considered against the mean, rendering the resultant distribution symmetrical on the *y*-axis. These differences from Wade et al. allowed for the generation of a distribution of replicate values, and hence the direct determination of variability.

#### Analytical variability

Analytical variability (AV) is related to the quantification step via RT-qPCR and it was assessed through a comparison of the concentration of each RT-qPCR technical replicate (TR) to the concentration of its corresponding biological replicate (BR) to describe continuous variability through the determination of the confidence range. The confidence range associated with analytical variability for each target is reported in Table 3.

**Table 3:** Analytical variability data for each target. We report the confidence range and the equivalent coefficient of variation (%)

Target	Confidence range (CR)	Coefficient of variation (%CV)
SARS-CoV-2 N1	3.12×	69.9%
SARS-CoV-2 E-Sarbeco	3.86×	89.6%
Total Estimated SARS-CoV-2	4.13×	72.1%
CrAssphage	4.11×	96.3%

The %CV results were within the range reported by Taylor et al. for general RT-qPCR variability and below the upper bound of 200% [37]. Although high variabilities may be unavoidable, their impacts could be reduced through the application of a large number of technical replicates: [36] this may be prohibitively expensive, however. Variability typically decreased in the order CrAssphage > E-Sarbeco > N1 between the RT-qPCR targets, with discrete variability (prevalence non-detect %) and continuous variability (CR, %CV) following the same trend. It can be observed in Figures S4-6(c), that the probability of a technical replicate being a non-detect decreased with increasing biological replicate concentration: low sample concentration was a leading cause of non-detects, indicating that non-detects emerged from the same mechanisms that drove continuous variability, further indicating that non-detects were *missing not at random*, supporting the conclusions of McCall et al. [38] in their previous analysis of the causes for non-detects [38]. The spread of BR:TR deviation ratios in Figures S4-6(a) was largely concentration-independent: this indicated a consistent %CV throughout the range for each target, while the absolute variance differed with the magnitude of the biological replicate concentration. Furthermore, the spread of ratios in Figures S4-6(b and d) was found to approximate a normal distribution: from these values, the standard deviation (*σ*) and 95% confidence interval (*μ* ± 2*σ*) were estimated for all targets. All target datasets displayed in Figures S4-6 were long tailed compared to the normal distribution indicating that any given technical replicate may be significantly below/above the biological replicate concentration. This was likely primarily driven by stochastic variability of qPCR inhibition between technical replicates, in line with discussion of such variabilities by McCall et al. [38]. The long tails presented within all datasets reinforce the decision to use the non-detect-removed geometric mean for data processing within this methodology as an interim non-optimised compromise between retaining information and ensuring robustness to outliers. Such broad variability could not be entirely resolved at the data processing stage, but the use of the geometric mean with non-detect removal was an effective strategy to minimise the impact of outliers.

#### Processing variability

Processing variability (PV) describes the variability attributable to sample processing, including the variability from sampling, sample pre-treatment, sample concentration, and nucleic acid extraction. PV confidence ranges for gene targets included in this study are collated in Table 4.

**Table 4:** Processing variability data for each target, reporting confidence range, and the equivalent coefficient of variation (%)

Target	Confidence range (CR)	Coefficient of variation (%CV)
SARS-CoV-2 N1	2.72×	58.6%
SARS-CoV-2 E-Sarbeco	3.45×	78.8%
Total Estimated SARS-CoV-2	2.77×	59.9%
CrAssphage	8.32×	204.0%

PV yielded confidence ranges slightly lower than those from AV. It was observed that the probability of a biological replicate being non-detect (discrete variability) decreased with increasing DS replicate concentration, in line with Analytical Variability, although there was a relative reduction in the proportion of non-detect biological replicates as compared to technical replicates (Figs. S7-9(c)). This reflected the impact of non-detect removal, as only biological replicates comprising only non-detect technical replicates were defined as non-detects; therefore, the percentage of non-detects must decrease between technical replicates and the biological replicates they comprise. Moreover, the discrete PV typically decreased in the order CrAssphage > SARS-CoV-2 E-Sarbeco > SARS-CoV-2 N1 in line with the relative continuous variation for these three targets (represented by the confidence range and %CV), as described in Table 4 and similar to what observed for AV.

The relatively higher variability of E-Sarbeco as compared to N1 aligned with observations from other studies in the field. Zhang et al. noted that the SARS-CoV-2 E-Sarbeco target showed poor analytical sensitivity in wastewater, particularly as compared to SARS-CoV-2 N1, in contrast to the equivalent high performances of N1 and E-Sarbeco in spiked samples [17, 39]. It has been hypothesised that E gene variability in wastewater may be due to its variable loss in the clean-up procedure, potentially due to binding with viral proteins in the wastewater sample [40].

The high variability of CrAssphage made it unsuitable for use as an internal positive control and population biomarker. While AV for this target is in line with that observed for SARS-CoV-2, the PV was significantly higher. A confidence range of 8.32× indicated that a daily sample with a CrAssphage concentration of 1000 gc/L may exhibit biological replicate concentrations between 120 and 8320 gc/L: a range so wide as to be quantitatively inapplicable. In this context, it was unsurprising that WBE data normalisation with CrAssphage has been found to reduce the value of epidemiological information compared to un-normalised data [41, 42].

Using CrAssphage as a normalisation biomarker without a robust assessment of its variability within a catchment area (as conducted in this study) may introduce increased uncertainty in the resulting measurements, not due to the normalisation procedure per se, but from the inherent variability of CrAssphage [43, 44], affecting the ability to distinguish genuine detections from false negatives. This is crucial aspect to consider within outbreak detection monitoring programmes, where missed detections can lead to delayed responses and underestimation of public health risks; therefore the variability of normalisation approaches should be assessed to obtain a robust measurement to support timely and reliable decision-making.

PV was more likely caused by processing rather than sample collection; indeed, Ahmed et al*.* focused on assessing sampling variability for CrAssphage, HAdV, and PMMoV: on converting their data into confidence range terms, sample differences were only associated with approximately a 1.1× confidence range for CrAssphage and a 1.25× confidence range for HAdV on the most variable day [34]. Therefore, sample extraction and concentration were more likely the sources of variability: indeed, Wade et al. discussed the high variability of the concentration step, noting that recovery of φ6 phage (a concentration and extraction control) may range from 1 to 50% [36]. Assuming these recoveries were 5th and 95th percentile values, they were analogous to a confidence range of 7.1×, with a similar magnitude of variability as that observed for CrAssphage in this study [36], exceeding that observed for SARS-CoV-2 N1 and E-Sarbeco.

#### Methodological variability

Methodological variability (MV) describes the overall variability inherent in the method and could be considered as the combined expression of both AV and PV. As previously observed for both AV and PV, the spread of DS:TR concentration ratios in Figures S10-12(a) was largely concentration-independent (consistent %CV and confidence range); the spread of ratios in Figures S10-12 (b and d) approximated a normal distribution from these values; therefore, the standard deviation (σ), 95% confidence interval (*μ* ± 2*σ*), and confidence range (2*σ*) were estimated for all targets. These figures are reported in both Table 5 and Figures S10-12 (a, b, and d).

**Table 5:** Methodological variability data for each target, reporting confidence range, and the equivalent coefficient of variation (%)

Target	Confidence range (CR)	Coefficient of variation (%CV)
SARS-CoV-2 N1	4.18×	97.6%
SARS-CoV-2 E-Sarbeco	5.35×	127.1%
Total Estimated SARS-CoV-2	5.15×	92.8%
CrAssphage	11.57×	280.5%

In line with the AV and PV, discrete MV typically decreases in the order CrAssphage > E-Sarbeco > N1. This was broadly aligned with the relative MV for all RT-qPCR targets (represented by the confidence range and %CV), as described in Table 5. Within the total MV, a strong relationship was observed between gene concentration within a sample and the proportion of non-detects: DS with lower concentrations contained more TR and BR non-detects. As shown in Figures S10-12(c), the probability of a technical replicate being non-detect decreased with increasing DS replicate concentration. Although this was in line with both AV and PV, the proportion of non-detect replicates was higher in MV because this metric comprised both AV and PV, and thus the proportion was larger than either of these components individually. Within this analysis, non-detect observations did not seem to represent data *missing at random*, but rather data *missing not at random* dependent upon an unobserved gene expression within the daily sample itself [38]. Consequently, the removal of non-detects in the absence of corresponding partner replicates might lead to bias. The use of multiple TR and BR therefore provides a practical approach to partially mitigate bias caused by handling non-detects in RT-qPCR across different gene targets. Beyond our interim application of non-detect removal, further research is required for the development of optimal and pragmatic methodologies for non-detect handling for routine disease outbreak surveillance. The confidence range metric offered a more interpretable and coherent representation of variability for these data. The use of the confidence range was aligned with the best practice described by Taylor et al., wherein statistical analysis was carried out in ‘C_T_-space’ using normalised log-transformed data [37]. Although this approach was coherent and appropriate for data with symmetrical concentration-independent variability on a logarithmic scale, the use of the confidence range for RT-qPCR data in WBE represented a novel innovation, as most WBE studies rely on %CV for the characterisation of variability in RT-qPCR data. Alongside the confidence range, the percentage coefficient of variation (%CV) was reported for AV, PV, and MV; however, the %CV metric was not well suited to this data since the underlying variability is log-normally distributed, thus dispersion was most appropriately expressed as a multiplicative factor rather than an additive deviation around a mean (Supplementary Information). Since variability is multiplicative, a confidence range expressed on the log-scale could span several orders of magnitude. For example, 2*σ* equivalent to a 10× confidence range for analytical variability would imply that a TR measurement of 10 gc/µL would correspond to a BR concentration between 1 and 100 gc/µL with ca. 95% confidence, assuming no underlying bias. Under a linear-normal interpretation however, %CV values could readily exceed 100% [37]. The same 10× spread would translate to a %CV of ca. 250%, which misleadingly would suggest that values could fall below zero (e.g. between −40 and 60 gc/µL for a measurement of 10 gc/µL). In reality, under the log-normal model, the plausible range was strictly positive and would span 1–100 gc/µL. For this reason, %CV values, while provided for reference, would be less informative than the confidence range metric for multiplicative variability.

### Assessing performance of composite metric for improved disease monitoring

SARS-CoV-2 N1 and E gene targets were both indicative of SARS-CoV-2 viral concentration. Therefore, the total SARS-CoV-2 viral concentration for each sample could be estimated using a weighted average of the available measurements. The authors were not aware of anyone else who had taken the step of merging WBE targets, except for Nagelkerke et al. who discussed a similar method for generating composite data in which data from consecutively analysed standard curves were merged to create a composite metric for the better estimation of SARS-CoV-2 concentration [45]. Nagelkerke found that this approach better mitigated stochastic error in RT-qPCR data for the proposed better prediction of COVID-19 caseload [45]; similar reduction in random error from the contributing gene targets was attempted in this work through the preparation of the Total Estimated SARS-CoV-2 metric. Final weights of 62.2:37.8 for SARS-CoV-2 N1 and E-Sarbeco were used respectively, derived from calculated %CV values for each target (Table 5).

Weights were defined as the inverse of the squared coefficient of variation ((1/%CV)^2^ = *μ*^2^/σ^2^) reducing bias towards lower concentrations, while giving a greater weight to the less variable N1 target as compared to E-Sarbeco. Though unusual, the use of %CV was in line with best practice as it was grounded in the statistics of the dataset [46]. However, two considerations influenced the weighting of N1 and E-Sarbeco: although the E gene exhibited greater variability and therefore warranted a lower weight, it was also observed at lower concentrations than N1 and would otherwise require scaling for comparability. The weighting approach prioritised adjusting for variability, although this may not be fully optimal given the opposing consideration of differential target expression rates.

The application of the composite metric Total Estimated SARS-CoV-2 reduced the incidence of non-detect BR as compared to E-Sarbeco and N1 (discrete PV). The number of non-detect DS decreased from 19 (N1) and 120 (E-Sarbeco) to 16 for Total Estimated SARS-CoV-2, out of a total of 525 DS (Figs. S13-15). Although this was not a large reduction from N1, including measurements from multiple targets improved robustness to non-detects. Furthermore, combining both N1 and E-Sarbeco TR in the calculation of BR of Total Estimated SARS-CoV-2 reduced the likelihood of non-detect BR within any given DS concentration range (Figures S13-15(c)); for example, in DS within the concentration range 1–10 gc/µL, the proportion of non-detects was reduced from 17.9% (N1) and 31.3% (E-Sarbeco) to 13.8%.

This showed that the composite metric Total Estimated SARS-CoV-2 preserved more information and robustness to non-detects than the individual gene targets alone, where non-detects were removed. However, using the composite metric led to an increase in the continuous processing variability (Figures S13-15a, b, d; Tables 6 and 7) as it was associated with the increase in TRs from one to two targets. As expected, the variability associated with an estimate of viral target concentration must be equal to or greater than the variability associated with the concentrations of its component gene targets. Thus, the increase in the DS:TR confidence range from 4.18× for SARS-CoV-2 N1 to 5.35× for Total Estimated SARS-CoV-2, in line with the confidence range of 5.15× for E-Sarbeco, could be seen as a consequence of the variability between the different gene target survival rates in wastewater, extraction rates in processing and detection rates in analysis. Figure S16 shows the differing performance of the N1 and E-Sarbeco targets at different concentrations. As such, the Total Estimated SARS-CoV-2 concentration for a BR would be between 125 and 165% of the measured value for an E-Sarbeco TR, while between 92 and 71% of the measured value for an N1 TR at low concentrations (1 gc/µL) and high concentrations (1 × 10^4^ gc/µL) respectively.

**Table 6:** Processing, and analytical and methodological variability values for Total Estimated SARS-CoV-2 composite metric

Type of variability	Confidence range (CR)	Coefficient of variation (%CV)
Analytical (AV)	4.13×	72.1%
Processing (PV)	2.77×	59.9%
Methodological (MV)	5.15×	92.8%

**Table 7:** Summary of continuous and discrete variability for all targets across AV, PV, and MV

Variability metric	Confidence range (continuous variability)			Non-detect probability (discrete variability)		
	Analytical (AV)	Process (PV)	Method (MV)	Analytical (AV)	Process (PV)	Method (MV)
SARS-CoV-2 N1	3.12×	2.72×	4.18×	11.5%	6.6%	17.1%
SARS-CoV-2 E-Sarbeco	3.86×	3.45×	5.35×	17.0%	16.6%	30.0%
Total Estimated SARS-CoV-2	4.13×	2.77×	5.15×	26.5%	5.2%	30.3%
CrAssphage	4.11×	8.32×	11.57×	4.3%	3.0%	7.1%

The composite metric Total Estimated SARS-CoV-2 therefore offered mixed benefits when applied to BR. It reduced the proportion of BR (and consequently DS) observations classified as non-detects, while yielding greater continuous variability in DS concentrations, reflecting differences in performance of gene targets. Overall, this composite metric provided a more robust relative measure for daily SARS-CoV-2 outbreak tracking than individual targets, although with reduced precision. Importantly, the structure of this composite metric allows the incorporation of additional gene targets to better track disease dynamics, suggesting potential applications as flexible approach for different targets beyond SARS-CoV-2.

### Limit of quantification (LOQ) and limit of detection (LOD)

The application of the ISO-11843 aligned approach to multiplicative variability is illustrated in Figure S17(a-b). Figure S17a shows the approach used within this manuscript visualised as the conventional additive formulation applied to log-transformed concentration data, where variability was treated as an additive normal error around zero (i.e. the LOD, 10^0^ gc/µL) and the LOQ was defined as ‘LOD + 1.64*σ*’, consistent with constant variability on the log-scale across the measurement range. Figure S17b presents this same approach visualised as the analogous multiplicative formulation applied to the linear concentration data. Here, variability was expressed on the log_10_ scale and the LOQ could be understood as being obtained through multiplying the LOD by the 95th percentile of the methodological variability (MV_95%_). Figure S17b displays the same distribution as in Figure S17a but plotted against the linear concentration axis to show how the additive ‘LOD + 1.64 *σ*’ logic was preserved when variability is multiplicative instead of additive. LOD and LOQ values calculated using this approach are reported in Table 8, alongside comparison LOD′ and LOQ′ values determined additively using the linear-adjusted standard deviation derived from Eq. 5.

**Table 8:** Limit of quantification (LOQ) for extracted nucleic acid (extr. NA) and wastewater samples for each target

	Log-scale		Linear adjusted		Log-scale		Linear adjusted	
	LOD	LOQ_MV95*%*_	′_3σ_	LOQ′_5σ_	LOD	LOQ_MV*95%*_	LOD′_3σ_	LOQ′_5σ_
	(gc/µL, extracted nucleic acid)				(× 10^3^ gc/L, wastewater)		(× 10^3^ gc/L, wastewater)	
SARS-CoV-2 N1	1.00	3.07	2.74	4.57	1.25	3.84	3.43	5.71
SARS-CoV-2 E-Sarbeco	1.00	3.97	3.87	6.44	1.25	4.96	4.84	8.05
Total Estimated SARS-CoV-2	1.00	3.72	3.56	5.93	1.25	4.65	4.45	7.41
CrAssphage	1.00	7.89	9.18	15.30	1.25	9.86	11.48	19.13

Values reported below the limit of quantification will typically originate from samples whose true concentrations lie within that range. However, assuming log-normal distributions, samples with true concentrations above the LOQ may be under-quantified into this range for < 5% of values, while samples with true concentrations below the LOD may be over-detected into this range for < 5% of values. Given the limited standardisation in WBE for the calculation of LOD/LOQ values from RT-qPCR data, LOD was defined as the non-detect cut-off (bottom calibrator) and the LOQ_MV95%_ values reported in Table 8 were thus broadly aligned with LOD values reported by others in the field for SARS-CoV-2. [47] reported an analytical LOD of 1.7 gc/μL and 3.8 gc/μL for SARS-CoV-2 N1 and SARS-CoV-2 E-Sarbeco, respectively, determined from spiked controls. These values were lower than the ones we report, but their means of determination indicate that they likely corresponded to approximately LOD_90_ instead of LOD_95_ [47]. Conversely, Ahmed et al. reported higher values, providing process LOD_95_ values of 7.24 × 10^4^ gc/L and 1.59 × 10^5^ gc/L for SARS-CoV-2 N1 and E-Sarbeco, respectively, determined from spiked controls [17]. Hassard et al. also reported process LOD values, although the method for their determination was not explicitly outlined: the values provided were 1.27 × 10^3^ gc/L and 2.13 × 10^4^ gc/L for SARS-CoV-2 N1 and E-Sarbeco [48]. Within the methodological framework proposed in this study, LOD/LOQ were defined by considering the methodological variability, providing a structured approach for interpreting low-concentration measurements and defining quantitative reporting limits for wastewater surveillance data.

Table 8 shows that the absolute difference between the LOD and LOQ was similar under both the log-scale approach and the linear-adjusted approach to which it was compared. However, the log-scale method better reflected the actual variability observed within the calibrated range and yielded statistically interpretable thresholds. In contrast, the comparative additive linear-adjusted method relied on a linear transformation of log-scale variability, which was inherently arbitrary. The resulting LOD depended entirely on how the log-scale standard deviation was rescaled: using the transforming the log-scale mean ± 2SD into linear form and dividing by four (per Table 8 and Eq. 5) produces one LOD value, whereas using the log-scale mean ± 0.5SD would produce a markedly lower one. Thus, the linear-adjusted approach yielded LOD and LOQ values that were sensitive to the chosen transformation but not as firmly anchored to the characteristics of the method, whereas the multiplicative log-scale approach provided a more coherent and defensible description of method performance.

## Conclusions

This manuscript provided a novel framework to characterise variability of WBE methods for disease outbreak tracking. The application of this methodology demonstrated that storage conditions strongly influenced the preservation of epidemiological signals in wastewater samples. Refrigeration at 4 °C maintained SARS-CoV-2 RNA stability and allowed to differentiate between samples across concentration ranges for up to seven days, whereas freezing at −20 °C and freeze-thaw cycling resulted in degradation and weaker correlations with clinical cases, resulting in a drop in precision from 97.7 to 33.3% and a reduction in Pearson’s *r* from 0.829 to −0.135 (SARS-CoV-2 N1) between frozen and refrigerated samples. The framework further enabled systematic quantification of variability across the analytical workflow, distinguishing contributions from RT-qPCR measurement (analytical variability), sample preparation (processing variability), and the entire workflow (methodological variability). Variability differed substantially between targets, highlighting the importance of target selection and demonstrating that some commonly used biomarkers (such as CrAssphage) may not always be suitable for normalisation and require further confirmation of their suitability. Finally, quantification of analytical and methodological variability allowed the definition of uncertainty bounds, of limits of quantification as applied to the native targets in wastewater, and the development of a composite signal metric integrating multiple assays to improve detection sensitivity. Together, these results demonstrated how structured evaluation of storage, variability, and quantification uncertainty could improve reliability and interpretation of WBE data for epidemiological surveillance.

## Supplementary Information

Below is the link to the electronic supplementary material.

ESM 1 Supplementary Material 1 (PDF 2.18 MB)

## Data Availability

The data supporting the findings of this study are available from the corresponding author upon reasonable request.
