# Supplementary material for: Accounting for methodological variabilities in disease outbreak surveillance using wastewater-based epidemiology: a case study with SARS-CoV-2
Source: Anal Bioanal Chem. 2026 Aug 8;418(21):7163–81. doi: 10.1007/s00216-026-06712-x (PMC13658344; doi:10.1007/s00216-026-06712-x)
Supplement: Supplementary file 1 [file 216_2026_6712_MOESM1_ESM.pdf]

SUPPLEMENTARY MATERIAL

Accounting for methodological variabilities in disease outbreak surveillance using wastewater-based epidemiology: a case study with SARS-CoV-2

James Boxall-Clasby[1,2,3], Nicola Ceolotto[1,2,3], Marjorie J. Gibbon[2,4], Kishore K. Jagadeesan[1,2], Edward J Feil[2,3,4,5], Theresa Smith[2,6], Megan Robertson[2,7], Richard Standerwick[8], Ruth Barden[7], Julian Faraway[2,6], Barbara Kasprzyk-Hordern[1,2,3]

Affiliations

1. Department of Chemistry, University of Bath, Bath, BA2 7AY
2. Centre of Excellence in Water-Based Early Warning Systems for Health Protection, University of Bath, Bath, BA2 7AY, UK
3. Institute for Sustainability, University of Bath, Bath, BA2 7AY, UK
4. Milner Centre for Evolution, Department of Life Sciences, University of Bath, Bath, BA2 7AY
5. Centre for Mathematical Biology, University of Bath, Bath, BA2 7AY, UK
6. Department of Mathematical Sciences, University of Bath, Bath, BA2 7AY
7. Wessex Water, Bath, BA2 7WW, UK
8. Environment Agency, Horizon House, Deanery Road, Bristol, UK

Author for correspondence: Nicola Ceolotto (nc819@bath.ac.uk)

Table of Contents

Page 3 **SARS-CoV-2 Reference Sequences**

Page 4 **UKHSA COVID-19 Incidence Data**

Page 5 **Storage Stability**

Fig. S1(a-c). Stability trends for of SARS-CoV-2 E-Sarbeco (a) and N1 (b) targets recovered from replicates of a wastewater samples stored at 4°C reported as Log-difference. For each time point, prevalence of non-detects (as percentage) and standard deviation (log) were reported.

Page 6 Fig. S2(a-c). Stability trends for of SARS-CoV-2 targets E-Sarbeco (a) and N1 (b) of wastewater replicates kept frozen (-20°C) as Log-difference. For each time point, prevalence of non-detects (as percentage) and standard deviation (log) were reported.

Page 7 Fig. S3(a-c). Stability of SARS-CoV-2 E-Sarbeco (a) and N1 (b) targets in wastewater replicates after multiple freeze-thaw cycles as Log-difference. For each time point, prevalence of non-detects (as percentage) and standard deviation (log) were reported.

Page 8 **Variability Characterisation**

Analytical Variability

Fig. S4(a-c) Visualisation of Analytical Variability (AV) for the SARS-CoV-2 E-Sarbeco gene target.

Fig. S5(a-c) Visualisation of Analytical Variability (AV) for the SARS-CoV-2 N1 gene target.

Page 9 Fig. S6(a-c) Visualisation of Analytical Variability (AV) for the CrAssphage gene target.

Processing Variability

Fig. S7(a-c) Visualisation of Processing Variability (PV) for the SARS-CoV-2 E-Sarbeco gene target.

Page 10 Fig. S8(a-c) Visualisation of Processing Variability (PV) for the SARS-CoV-2 N1 gene target.

Fig. S9(a-c) Visualisation of Processing Variability (PV) for the CrAssphage gene target.

Page 11 **Methodological Variability**

Fig. S10(a-c) Visualisation of Methodological Variability (MV) for the SARS-CoV-2 E-Sarbeco gene target.

Fig. S11(a-c) Visualisation of Methodological Variability (MV) for the SARS-CoV-2 N1 gene target.

Page 12 Fig. S12(a-c) Visualisation of Methodological Variability (MV) for the CrAssphage gene target.

Total Estimated SARS-CoV-2 Composite Metric

Fig. S13(a-c) Visualisation of Analytical Variability (AV) for Total Estimated SARS-CoV-2 composite metric.

Page 13 Fig. S14(a-c) Visualisation of Processing Variability (PV) for Total Estimated SARS-CoV-2 composite metric.

Fig. S15(a-c) Visualisation of Methodological Variability (MV) for Total Estimated SARS-CoV-2 composite metric.

Page 14 Fig. S16(a-b) Visualisation of (a) Processing Variability (PV) and (b) Methodological Variability (MV) for Total Estimated SARS-CoV-2 composite metric, identifying TR data originating from SARS-CoV-2 E-Sarbeco and N1 gene targets.

Page 14 Fig. S17(a-b) Determination of LOQ from the LOD using (a) additive model for the determination of LOQ, applied to log-transformed concentration data, and (b) additive model visualised on the linear scale. The LOQ is the result of a mathematically analogous transformation, whereby the LOD was multiplied by the 95th percentile of the Methodological Variability ($MV_{95\%}$).

SARS-CoV-2 Reference Sequences

US CDC N1 (2019-nCoV N1)

Nucleotides 28287-28358 (N gene) of the Severe Acute Respiratory Syndrome Coronavirus 2 isolate Wuhan-Hu-1, NCBI Reference Sequence: NC 045512.2, as per work carried out by Vogels et al. (2020).

```
28287       GACC CCAAAAUCAG CGAAAUGCAC CCCGCAUUAC GUUUGGUGGA

28331 CCCUCAGAUU CAACUGGCAG UAACCAGA
```

Charité E (E-Sarbeco)

Nucleotides 26269-26381 (E gene) of the Severe Acute Respiratory Syndrome Coronavirus 2 isolate Wuhan-Hu-1, NCBI Reference Sequence: NC 045512.2, as per work carried out by

```
15431 GUGAAAUGGU CAUGUGUGGC GGUUCACUAU AUGUUAAACC AGGUGGAACC
15481 UCAUCAGGAG AUGCCACAAC UGCUUAUGCU AAUAGUGUUU UUAACAUUUG
```

Standard (Synthetic ds DNA fragment for quantification)

519 nt custom DNA fragment with target sequences for all four primer/probe sets (supplied by Eurofins). Underlined bases show forward primer, probe, reverse primer for crAssphage, SARS-CoV-2 N1, SARS-CoV-2 E-Sarbeco respectively. Intervening sequences are the same as the intervening sequences in the target genomes.

ACTGCTAATG**CAGAAGTACAAACTCCTAAAAAACGTAGAG**GTAGAGGTATT**AATAACGATTTACGTGATGTAAC**TCGTAAA
AAGTTTGATGAACGTACTGATTGTAATAAA**GCTAATGGCTTGTTTATTGGTCATC**TTGAATAATG**GACCCCAAAATCAGCG**
AAATGC**ACCCCGCATTACGTTTGGTGGACC**CT**CAGATTCAACTGGCAGTAACCAGA**ATGGAAAGAG**ACAGGTACGTTAATA**
GTTAATAGCGTACTTCTTTTTCTTGCTTTCGTGGTATTCTTGCTAGTT**ACACTAGCCATCCTTACTGCGCTTCG**AT**TGTGT**
GCGTACTGCTGCAATATTGTTAAGTACCAGGACTTCGGAGCCTCGTGCTGGGCGGCAAACGAGCTGTTAAACGAGGAGTGG
TTAACCTCGTCAAGTATGGCCGGTAAAAATCAGAGCCAGAAGATTAAGAAGAATACAGCTCCGATGGGGAATGGCCAGCCA
GTCAATCAACTGTGCCAGTTGCTGGGTGCAATG

UKHSA COVID-19 Incidence Data

Weekly UKHSA-reported COVID-19 incidence data was obtained for each Saturday within the study period,182 describing the number of COVID-19 cases confirmed within the prior 7 days. Weekly data was used to prevent the intra-week variability in UKHSA data from confounding the results. Data were obtained from the UK Government COVID-19 dashboard (https://coronavirus.data.gov.uk/) at the scale of Middle Layer Super Output Areas (MSOAs): MSOAs represent the finest scale for which COVID-19 case data is publicly available, and each MSOA typically has between 5,000 and 15,000 inhabitants. MSOA and wastewater catchment boundaries do not correspond to one another; a conversion based on geographical overlap was applied as per work previously carried out by Jagadeesan et al.88 This conversion is detailed in Equation 8 below.

$$y_{cw} = \sum_{m=1}^{M} \rho_{mc} y_{mw} \qquad (8)$$

Where y_{mw} represents the number of cases in catchment *c* in week *w*, ρ_{mc} the percentage of MSOA *m* in catchment *c*, and y_{mw} the number of cases in MSOA *m* in week *w*. Due to the nature of this calculation, non-integer COVID-19 case values are possible for a wastewater catchment, as well as values below 1. Additionally, in the original MSOA data, cases ≤ 2 are censored for patient confidentiality. In these situations, any surplus cases reported in the larger Local Authority region are divided equally between the contained censored MSOAs. Moreover, geographical overlap is not necessarily equivalent to population overlap: this discrepancy likely causes greater inaccuracy in the case data for the smaller catchments. Nevertheless, the calculated number of UKHSA-reported weekly COVID-19 cases is thought to be a good proxy for the true degree of COVID-19 outbreak. Due to differences in population density, MSOAs tend to be significantly smaller than wastewater catchment areas in urban areas, but of a more similar size in rural areas. Thus COVID-19 data from urban MSOAs can be unambiguously attributed to the encompassing wastewater catchment area, but the same is not necessarily the case for rural MSOAs. Indeed, none of the MSOAs associated with the Paulton catchment area are entirely encompassed by the Paulton wastewater catchment area. Thus, calculated wastewater catchment-equivalent COVID-19 case data may be less accurate in rural catchments due to boundaries mismatch; therefore, population mobility (e.g., using mobile phone data) and triangulation with multiple data sources could be integrated to minimize these differences in overlapping(1).

1. Jagadeesan KK, Robertson M, Bagnall J, Kasprzyk-hordern B. Demographic-Driven Wastewater-Based Epidemiology : Refining Population Estimates for Enhanced Chemical Monitoring. 2025;

Storage Stability

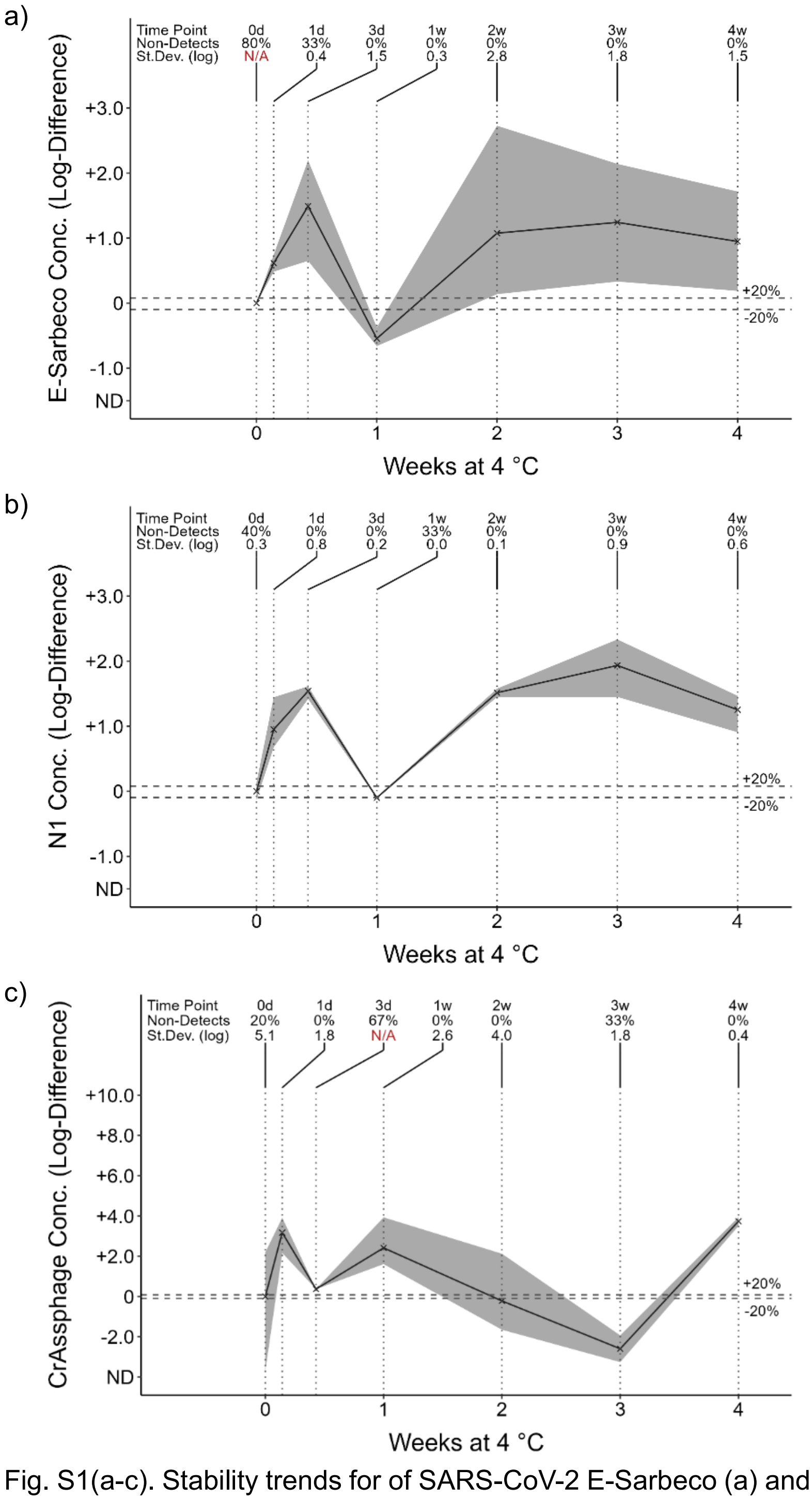

Fig. S1(a-c). Stability trends for of SARS-CoV-2 E-Sarbeco (a) and N1 (b) targets recovered from replicates of a wastewater samples stored at 4°C reported as Log-difference. For each time point, prevalence of non-detects (as percentage) and standard deviation (log) were reported.

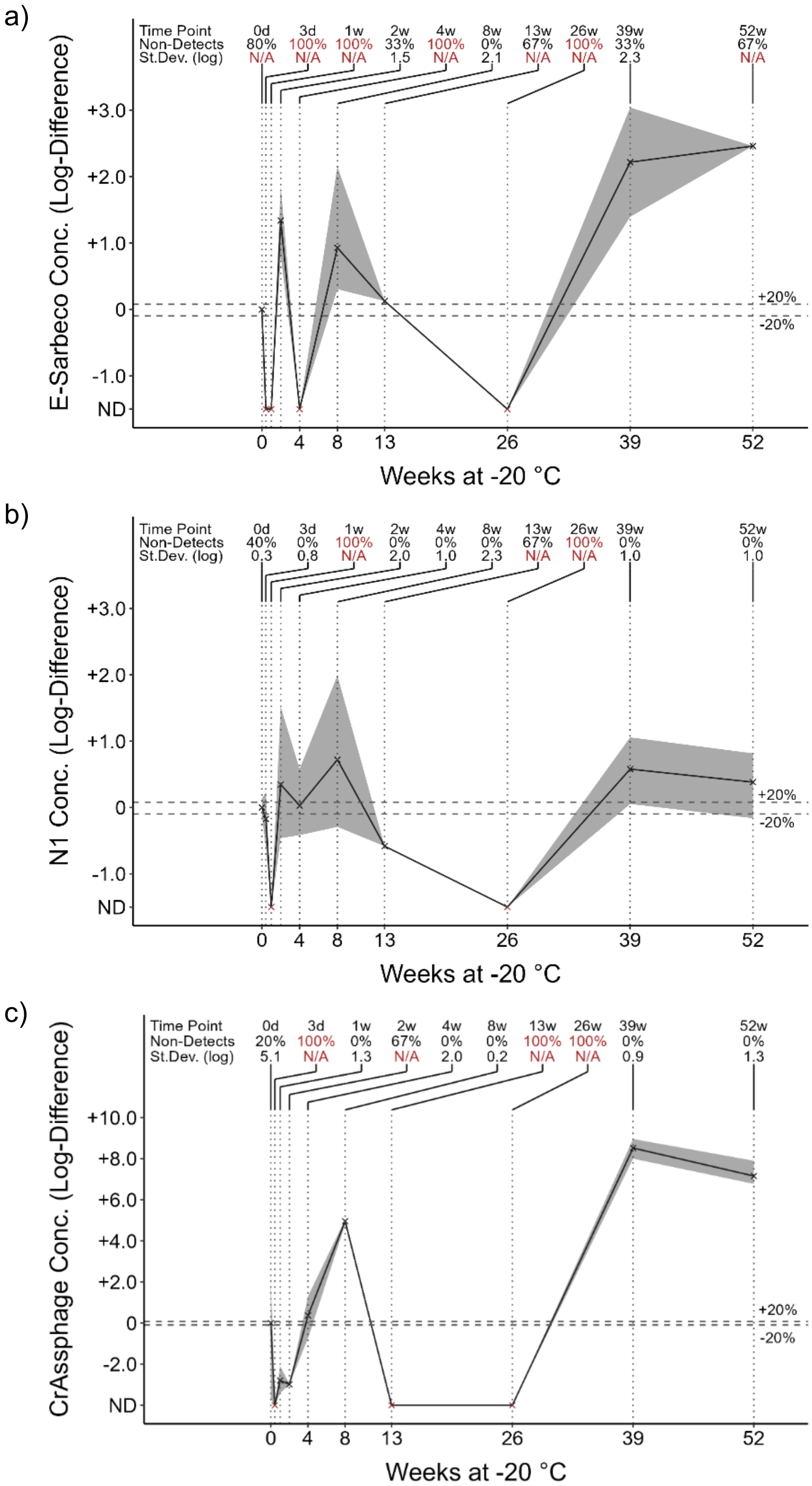

Fig. S2(a-c). Stability trends for of SARS-CoV-2 targets E-Sarbeco (a) and N1 (b) of wastewater replicates kept frozen (-20°C) as Log-difference. For each time point, prevalence of non-detects (as percentage) and standard deviation (log) were reported.

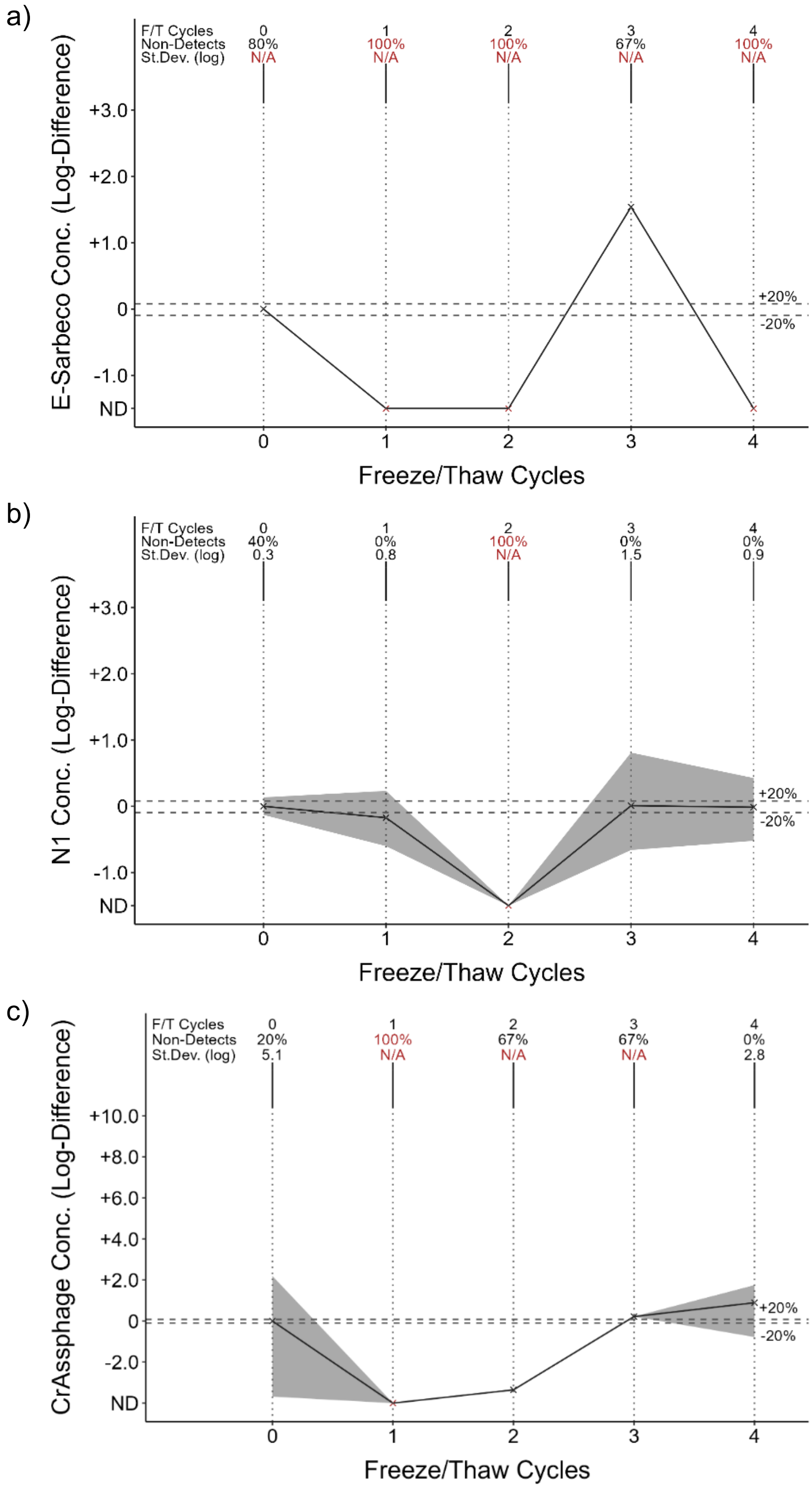

Fig. S3(a-c). Stability of SARS-CoV-2 E-Sarbeco (a) and N1 (b) targets in wastewater replicates after multiple freeze-thaw cycles as Log-difference. For each time point, prevalence of non-detects (as percentage) and standard deviation (log) were reported.

Variability Characterisation

Analytical Variability

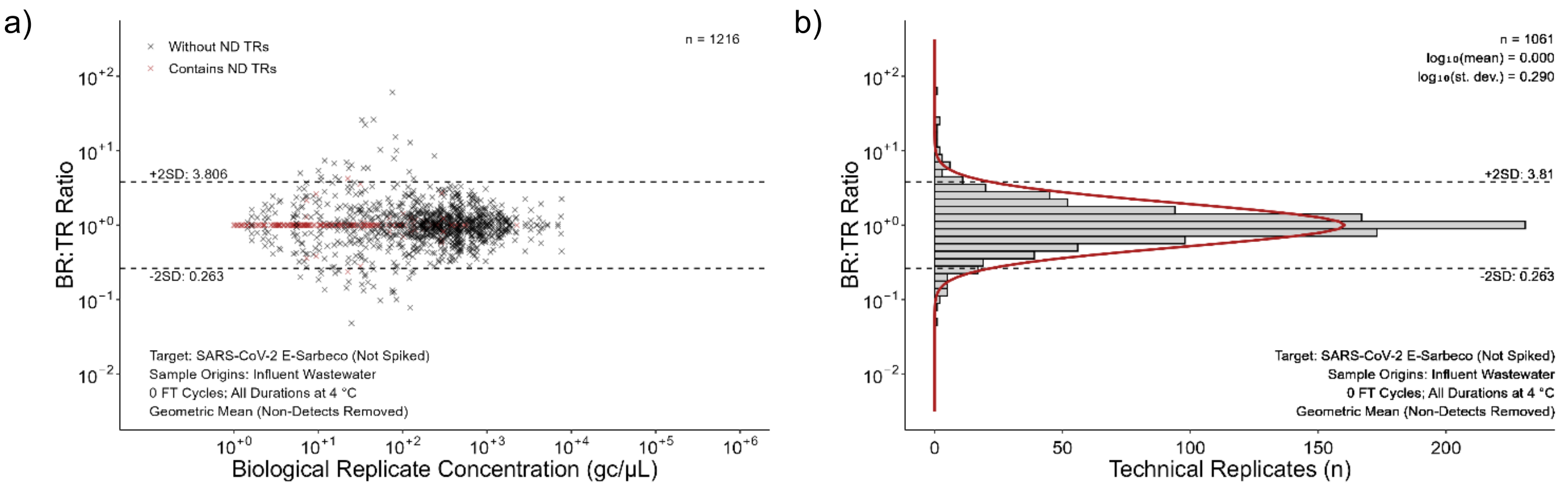

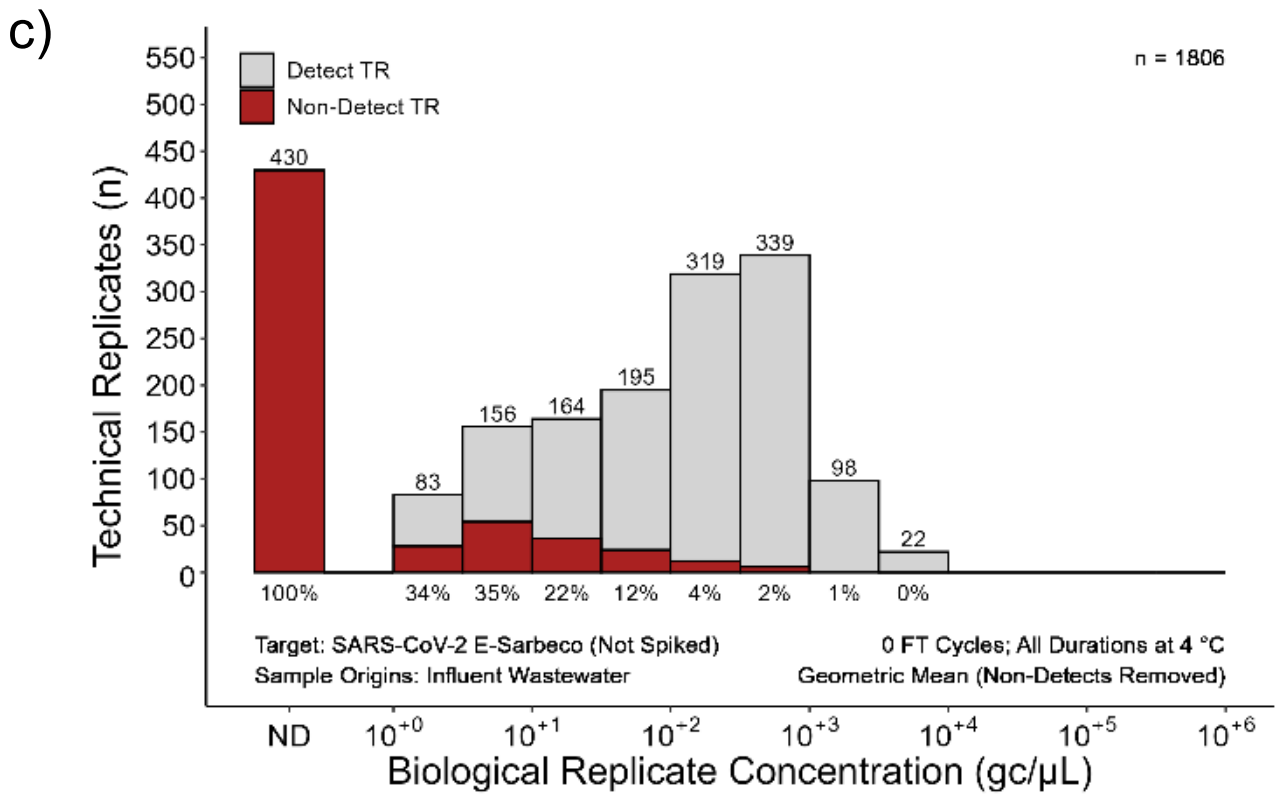

a) Continuous Analytical Variability for SARS-CoV-2 E-Sarbeco is visualised as a scatter plot of BR:TR ratio against BR concentration.

b) Continuous Analytical Variability for SARS-CoV-2 E-Sarbeco is visualised as a histogram of BR:TR ratio.

c) Discrete Analytical Variability for SARS-CoV-2 E-Sarbeco is visualised as a bar chart of detect/non-detect TRs against BR concentration range.

Fig. S4(a-c) Visualisation of Analytical Variability (AV) for the SARS-CoV-2 E-Sarbeco gene target.

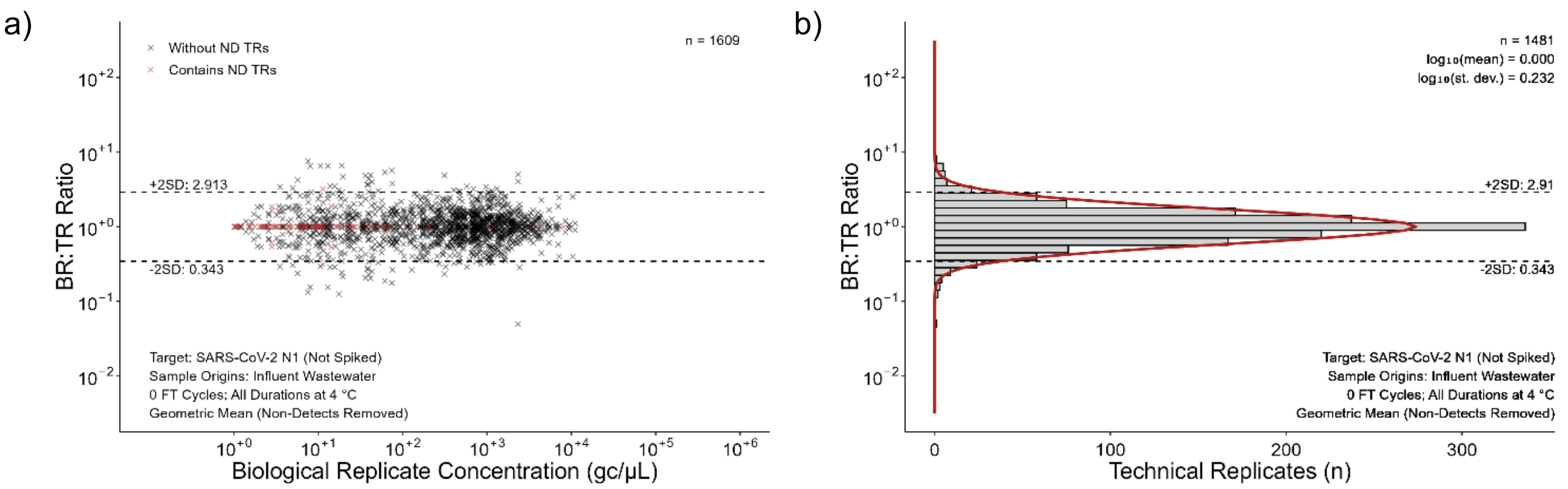

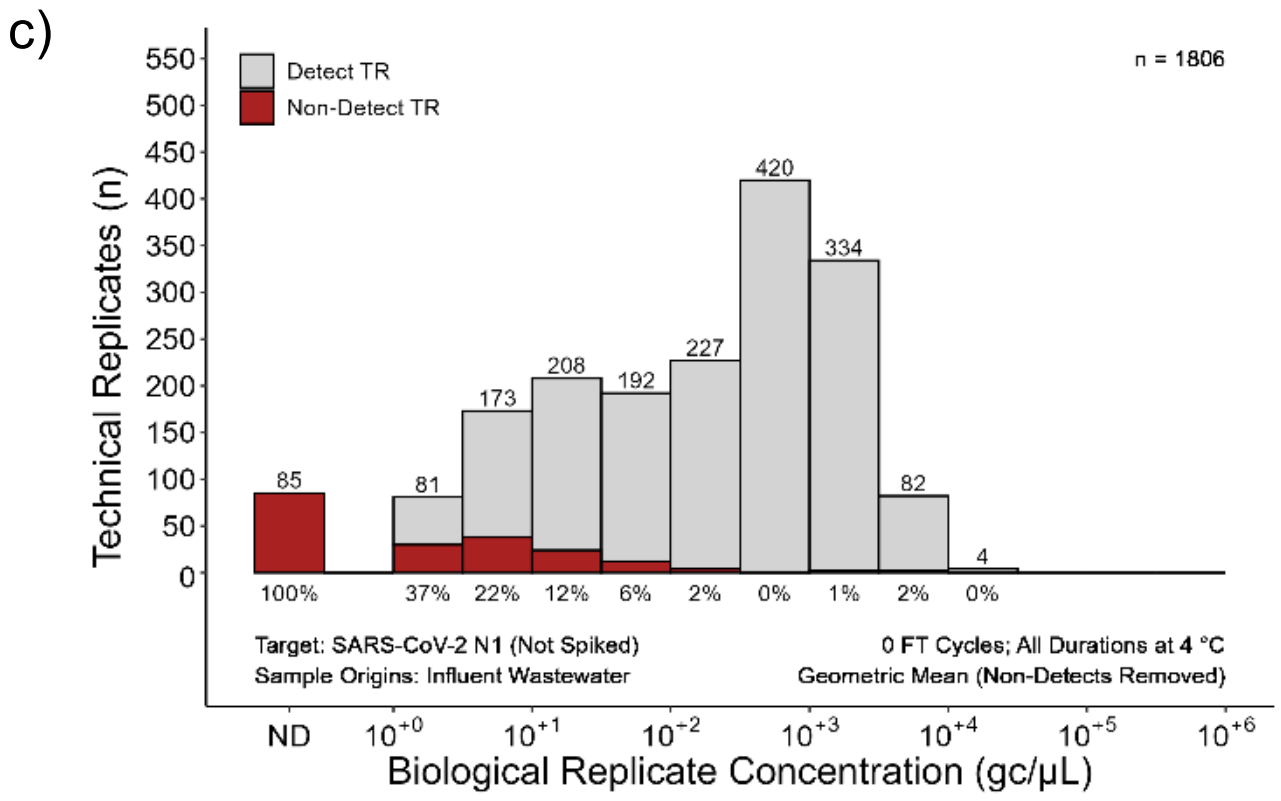

a) Continuous Analytical Variability for SARS-CoV-2 N1 is visualised as a scatter plot of BR:TR ratio against BR concentration.

b) Continuous Analytical Variability for SARS-CoV-2 N1 is visualised as a histogram of BR:TR ratio.

c) Discrete Analytical Variability for SARS-CoV-2 N1 is visualised as a bar chart of detect/non-detect TRs against BR concentration range.

Fig. S5(a-c) Visualisation of Analytical Variability (AV) for the SARS-CoV-2 N1 gene target.

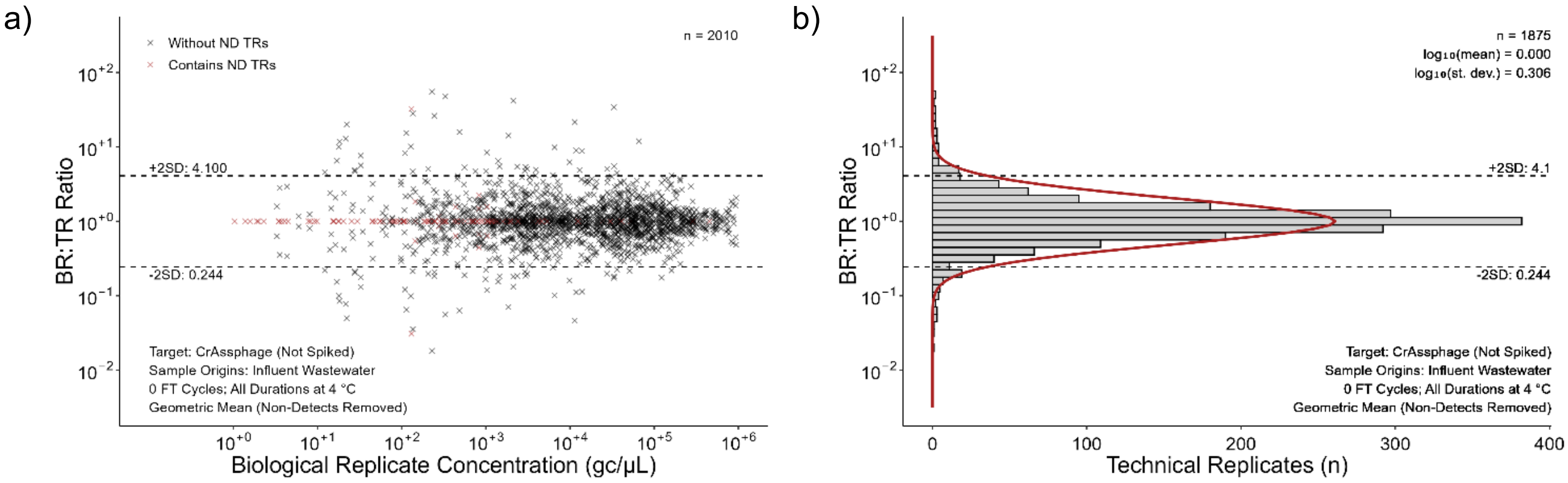

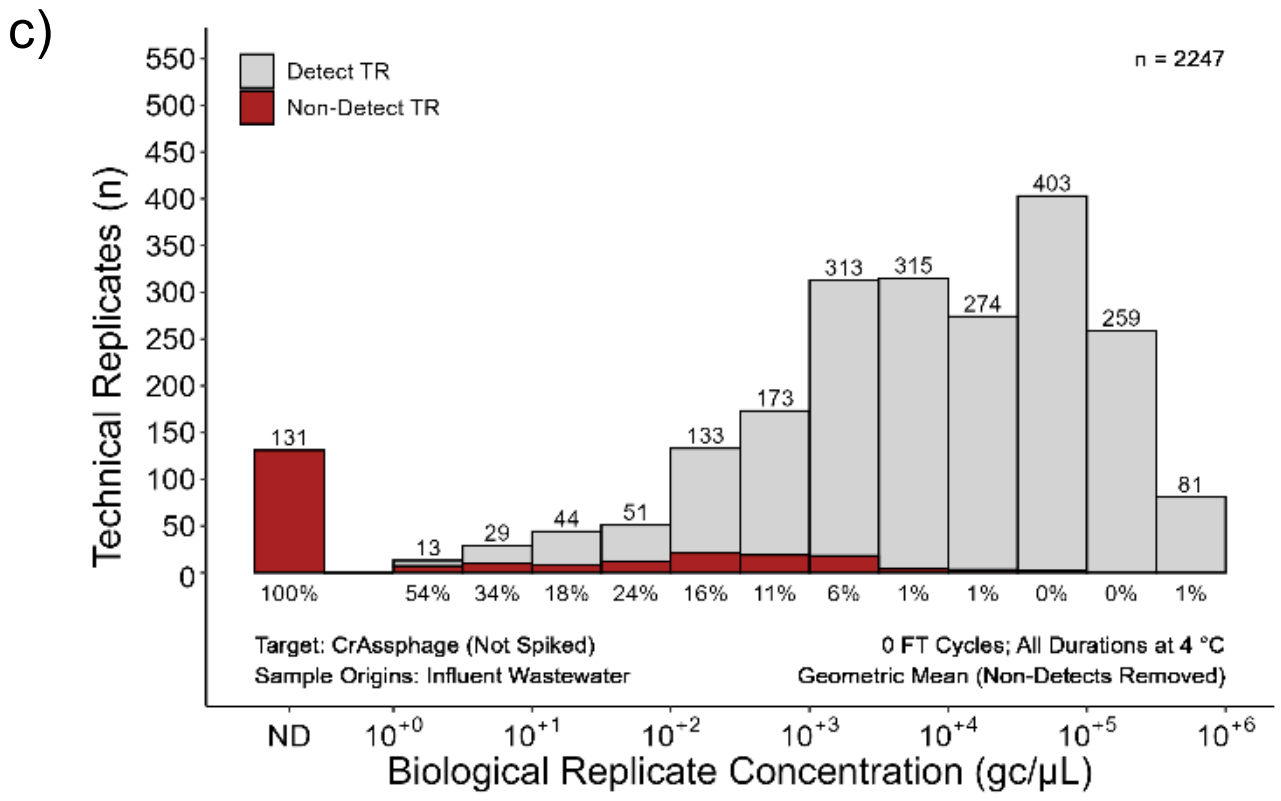

a) Continuous Analytical Variability for CrAssphage is visualised as a scatter plot of BR:TR ratio against BR concentration.

b) Continuous Analytical Variability for CrAssphage is visualised as a histogram of BR:TR ratio.

c) Discrete Analytical Variability for CrAssphage is visualised as a bar chart of detect/non-detect TRs against BR concentration range.

Fig. S6(a-c) Visualisation of Analytical Variability (AV) for the CrAssphage gene target.

Processing Variability

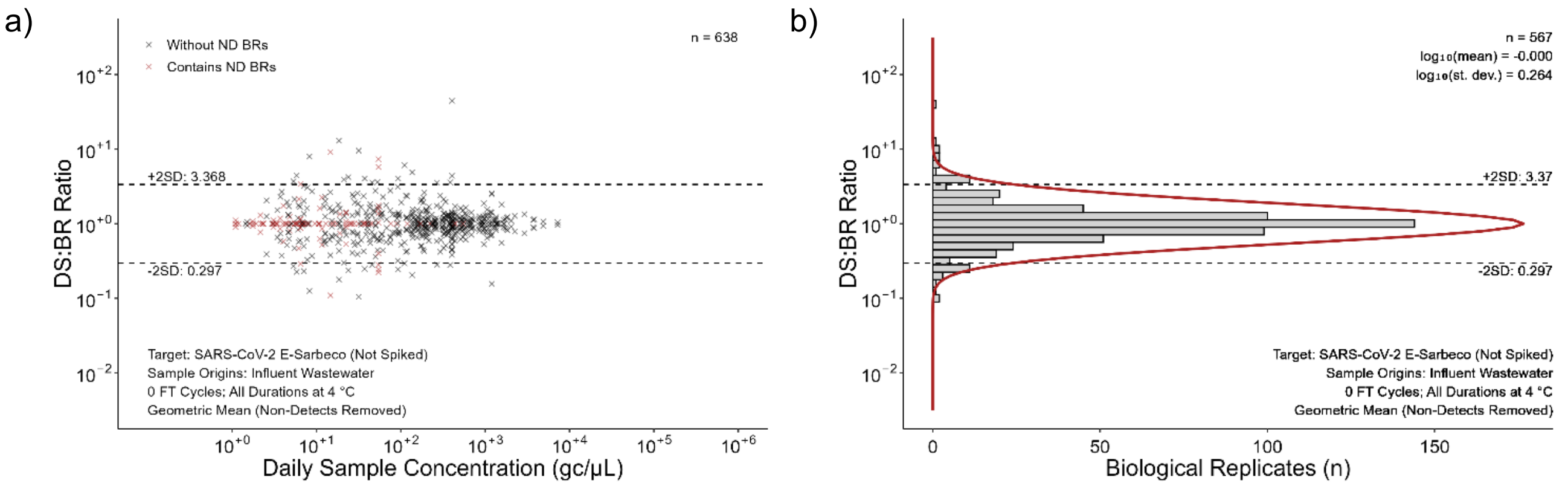

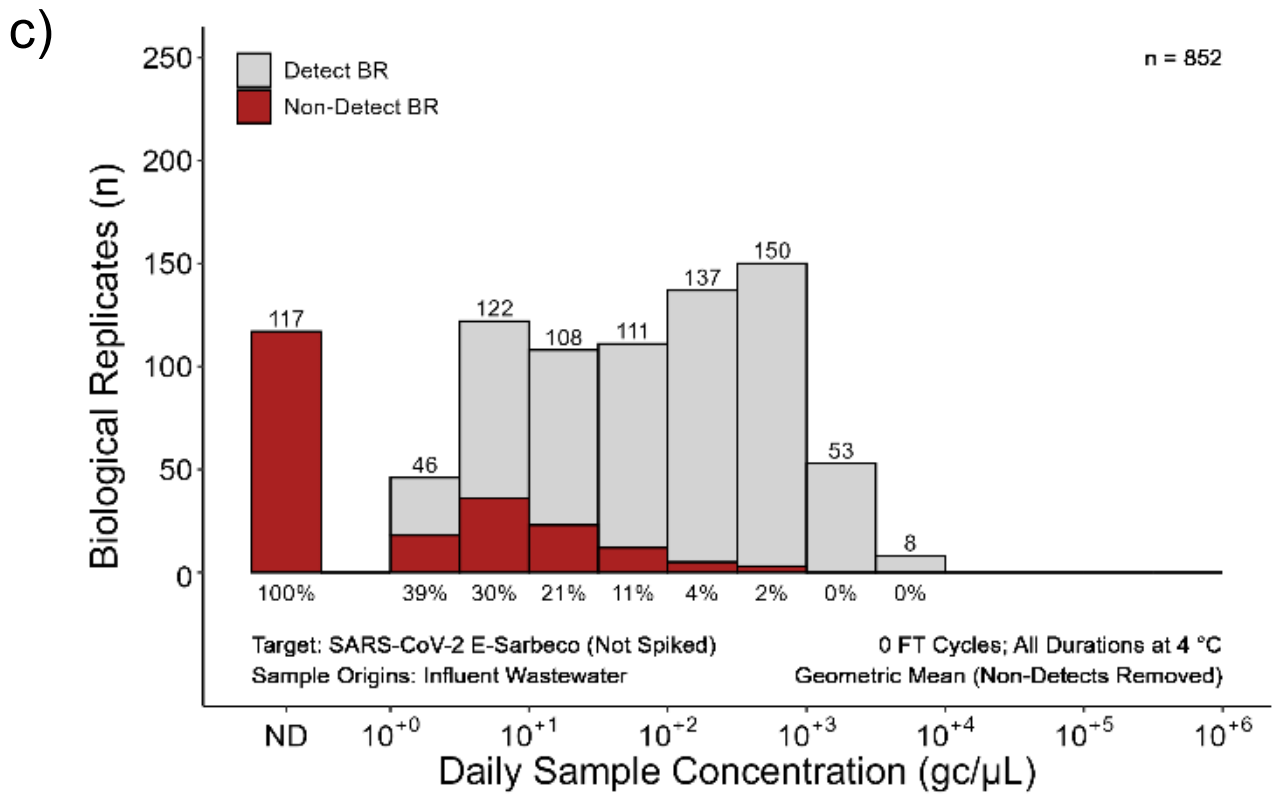

a) Continuous Processing Variability for SARS-CoV-2 E-Sarbeco is visualised as a scatter plot of DS:BR ratio against DS concentration.

b) Continuous Processing Variability for SARS-CoV-2 E-Sarbeco is visualised as a histogram of DS:BR ratio.

c) Discrete Processing Variability for SARS-CoV-2 E-Sarbeco is visualised as a bar chart of detect/non-detect BRs against DS concentration range.

Fig. S7(a-c) Visualisation of Processing Variability (PV) for the SARS-CoV-2 E-Sarbeco gene target.

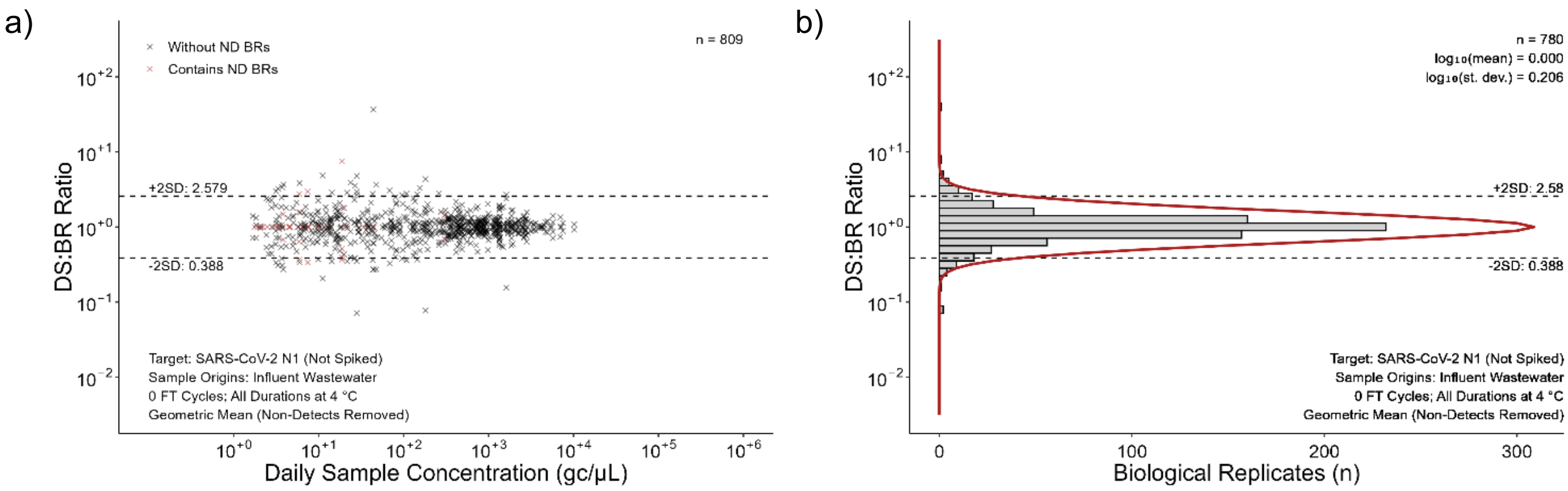

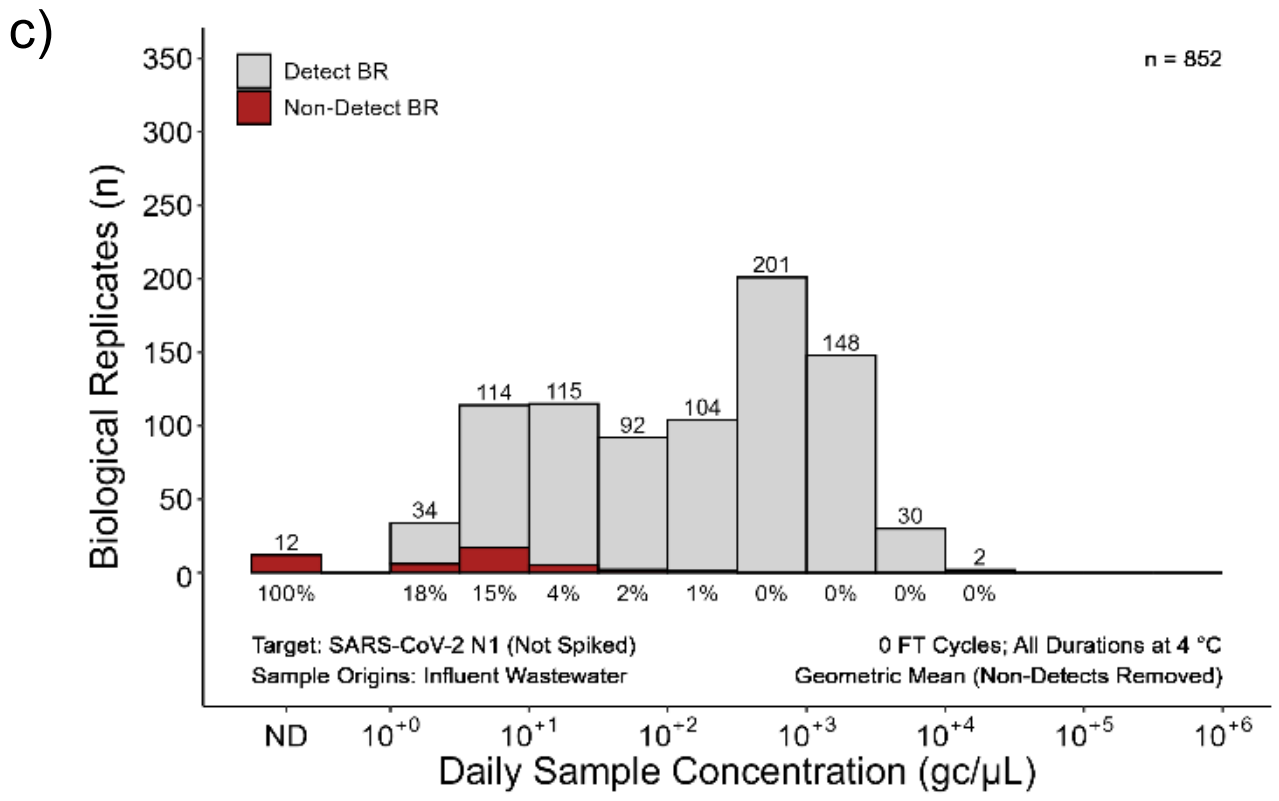

a) Continuous Processing Variability for SARS-CoV-2 N1 is visualised as a scatter plot of DS:BR ratio against DS concentration.

b) Continuous Processing Variability for SARS-CoV-2 N1 is visualised as a histogram of DS:BR ratio.

a) Discrete Processing Variability for SARS-CoV-2 N1 is visualised as a bar chart of detect/non-detect BRs against DS concentration range.

Fig. S8(a-c) Visualisation of Processing Variability (PV) for the SARS-CoV-2 N1 gene target.

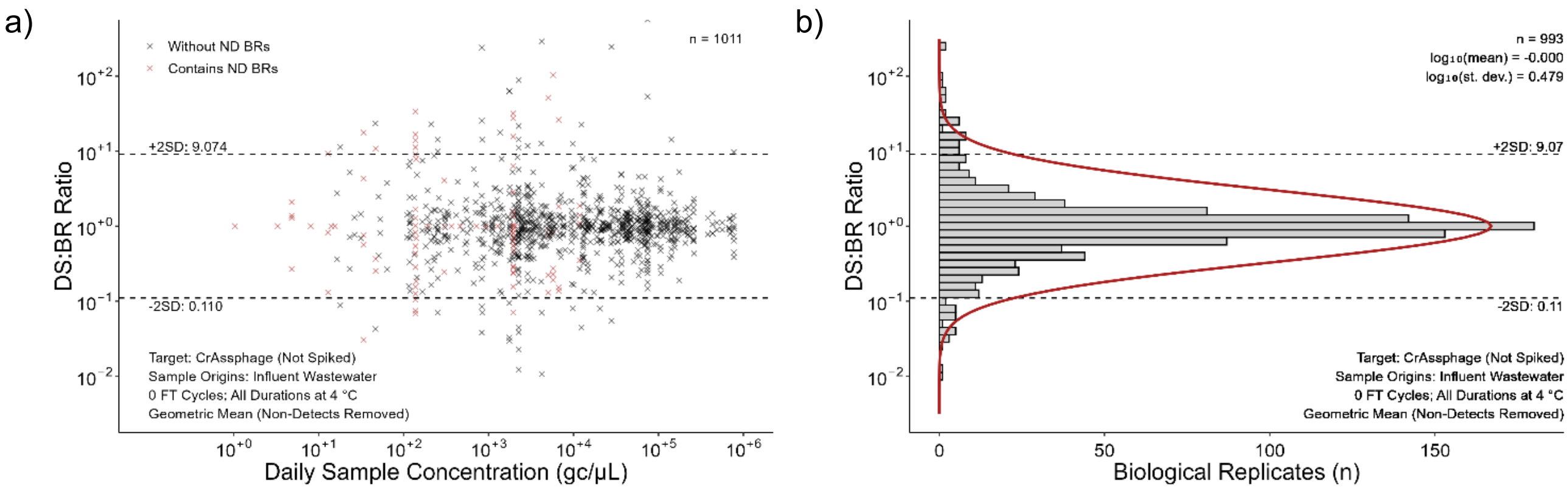

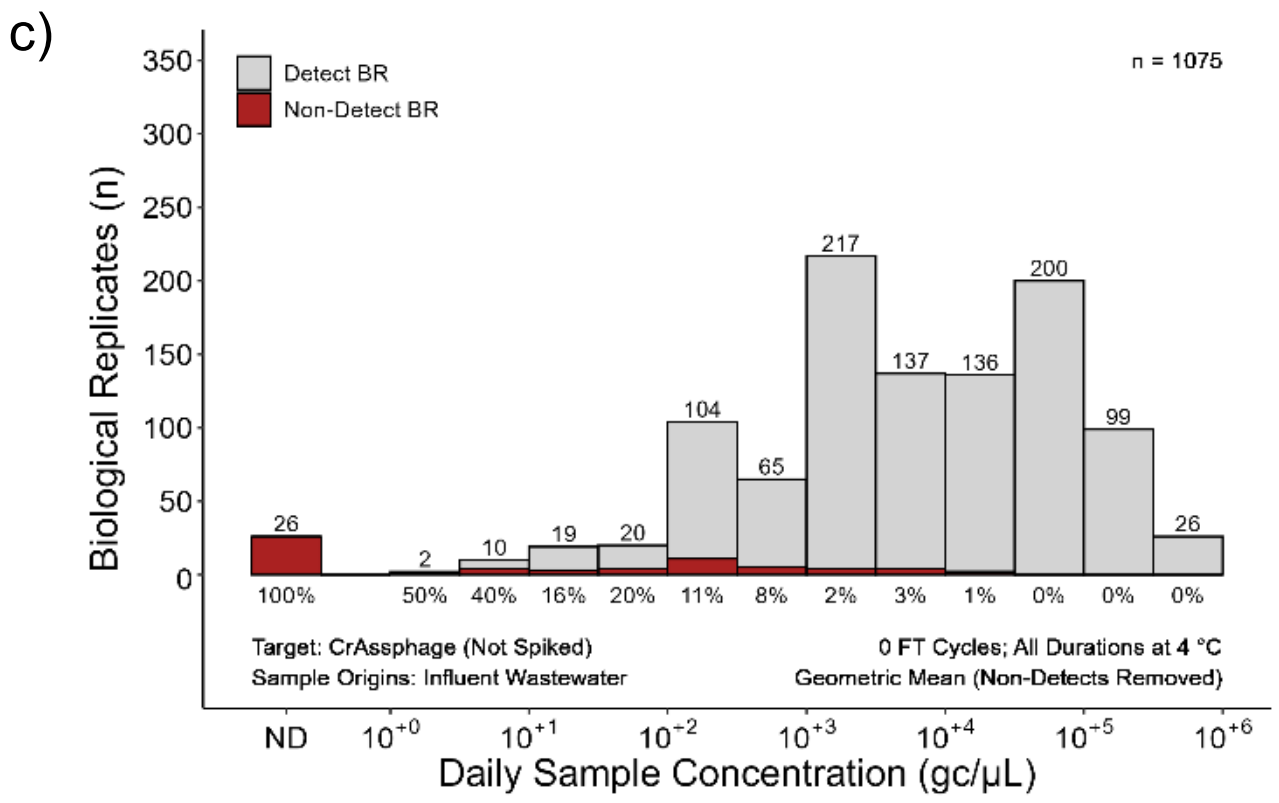

a) Continuous Processing Variability for CrAssphage is visualised as a scatter plot of DS:BR ratio against DS concentration.

b) Continuous Processing Variability for CrAssphage is visualised as a histogram of DS:BR ratio.

c) Discrete Processing Variability for CrAssphage is visualised as a bar chart of detect/non-detect BRs against DS concentration range.

Fig. S9(a-c) Visualisation of Processing Variability (PV) for the CrAssphage gene target.

Methodological Variability

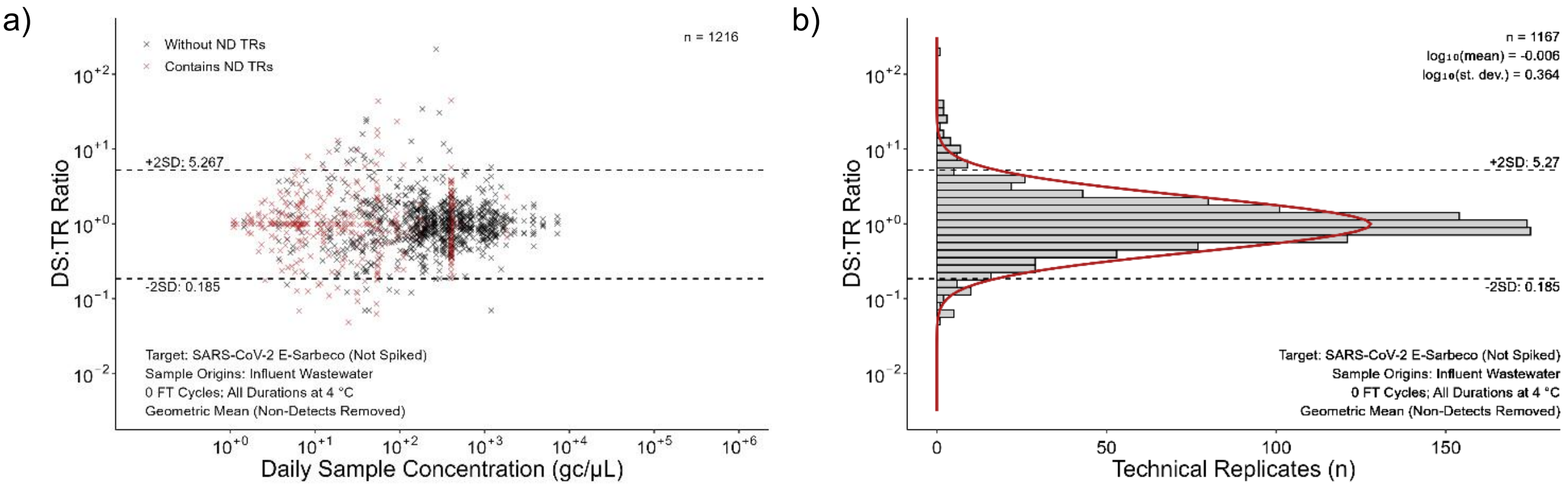

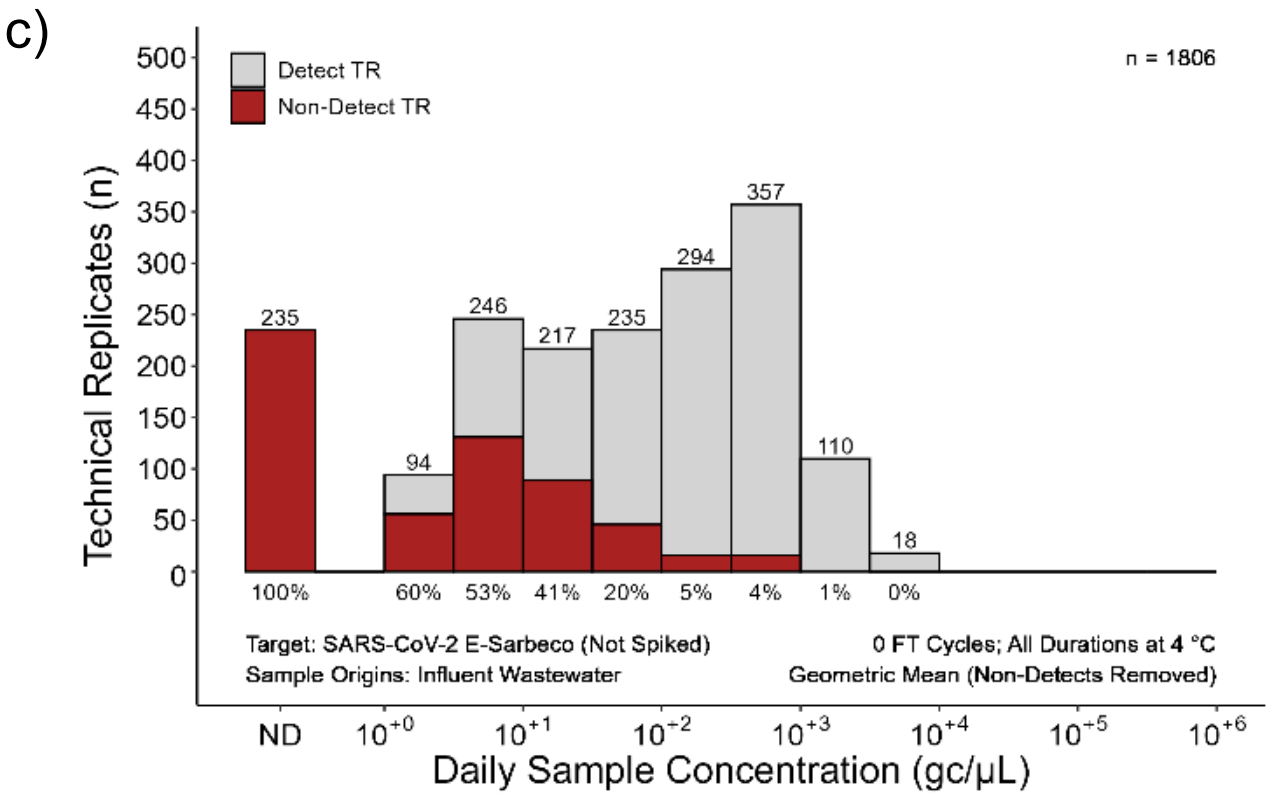

a) Continuous Methodological Variability for SARS-CoV-2 E-Sarbeco is visualised as a scatter plot of DS:TR ratio against DS concentration.

b) Continuous Methodological Variability for SARS-CoV-2 E-Sarbeco is visualised as a histogram of DS:TR ratio.

c) Discrete Methodological Variability for SARS-CoV-2 E-Sarbeco is visualised as a bar chart of detect/non-detect TRs against DS concentration range.

Fig. S10(a-c) Visualisation of Methodological Variability (MV) for the SARS-CoV-2 E-Sarbeco gene target.

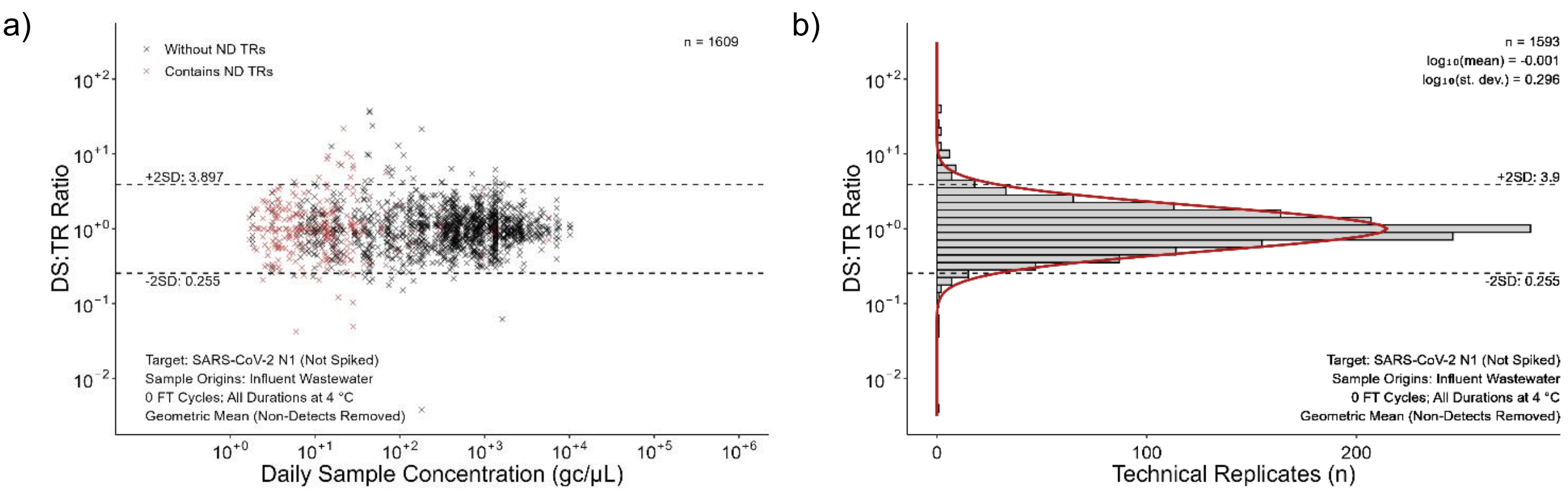

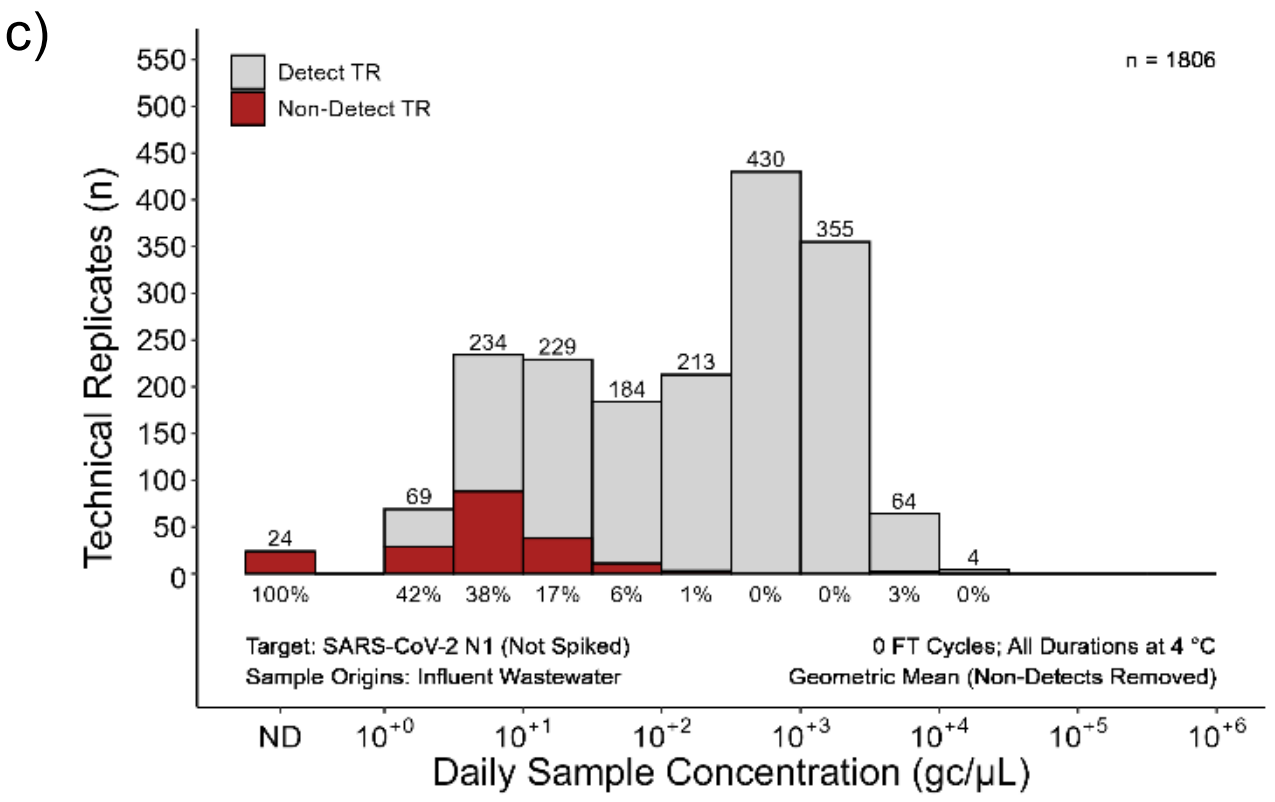

a) Continuous Methodological Variability for SARS-CoV-2 N1 is visualised as a scatter plot of DS:TR ratio against DS concentration.

b) Continuous Methodological Variability for SARS-CoV-2 N1 is visualised as a histogram of DS:TR ratio.

c) Discrete Methodological Variability for SARS-CoV-2 N1 is visualised as a bar chart of detect/non-detect TRs against DS concentration range.

Fig. S11(a-c) Visualisation of Methodological Variability (MV) for the SARS-CoV-2 N1 gene target.

a)

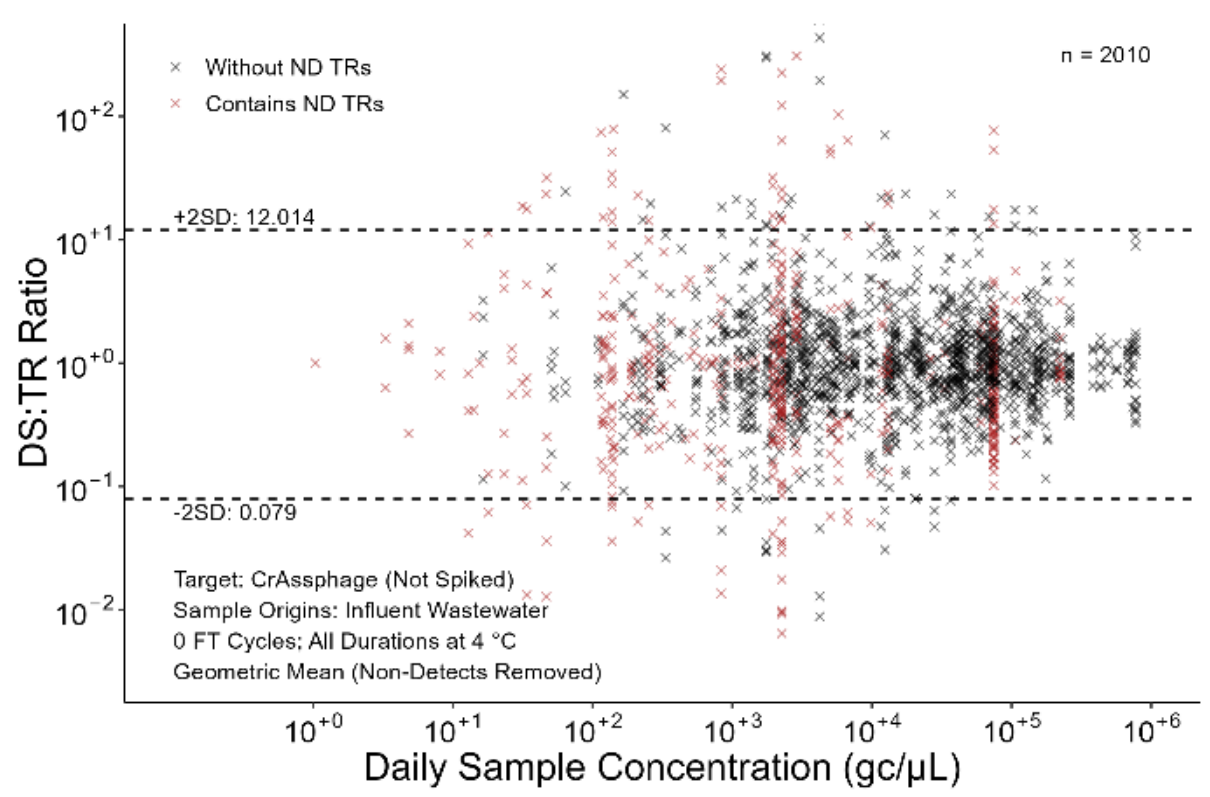

b)

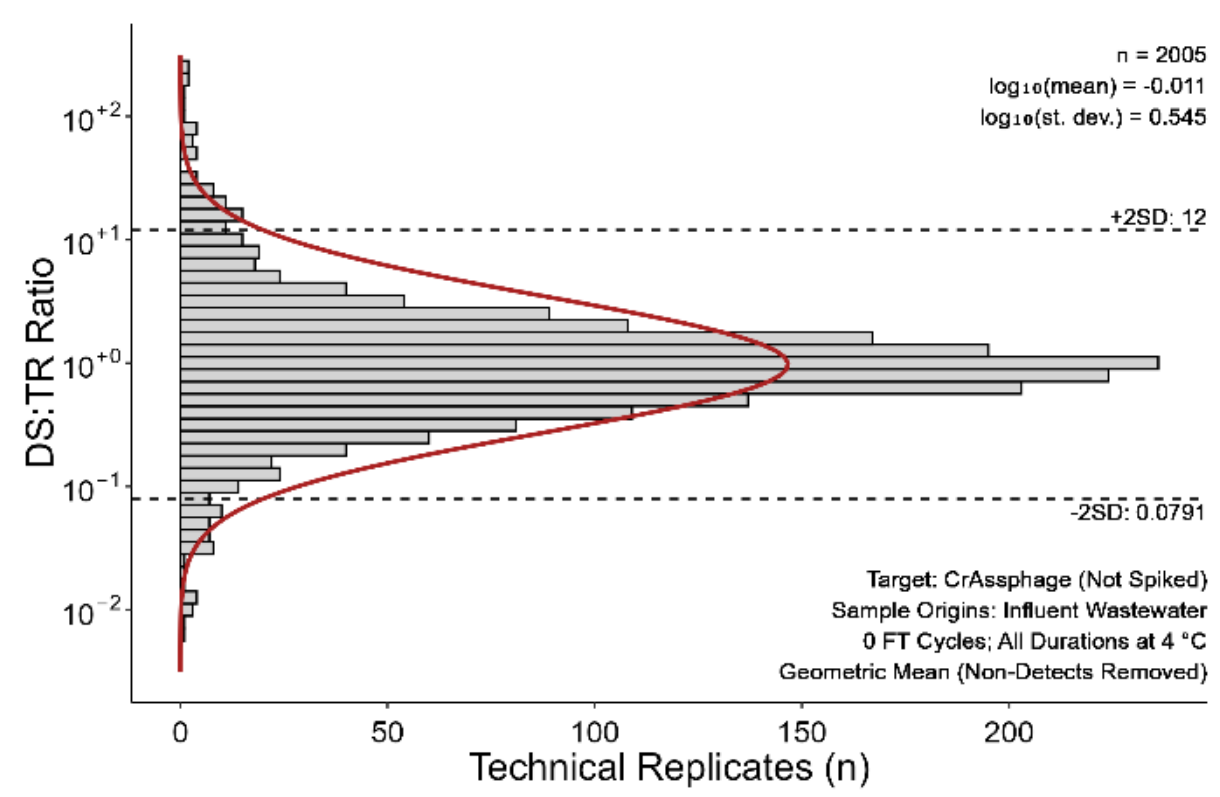

c)

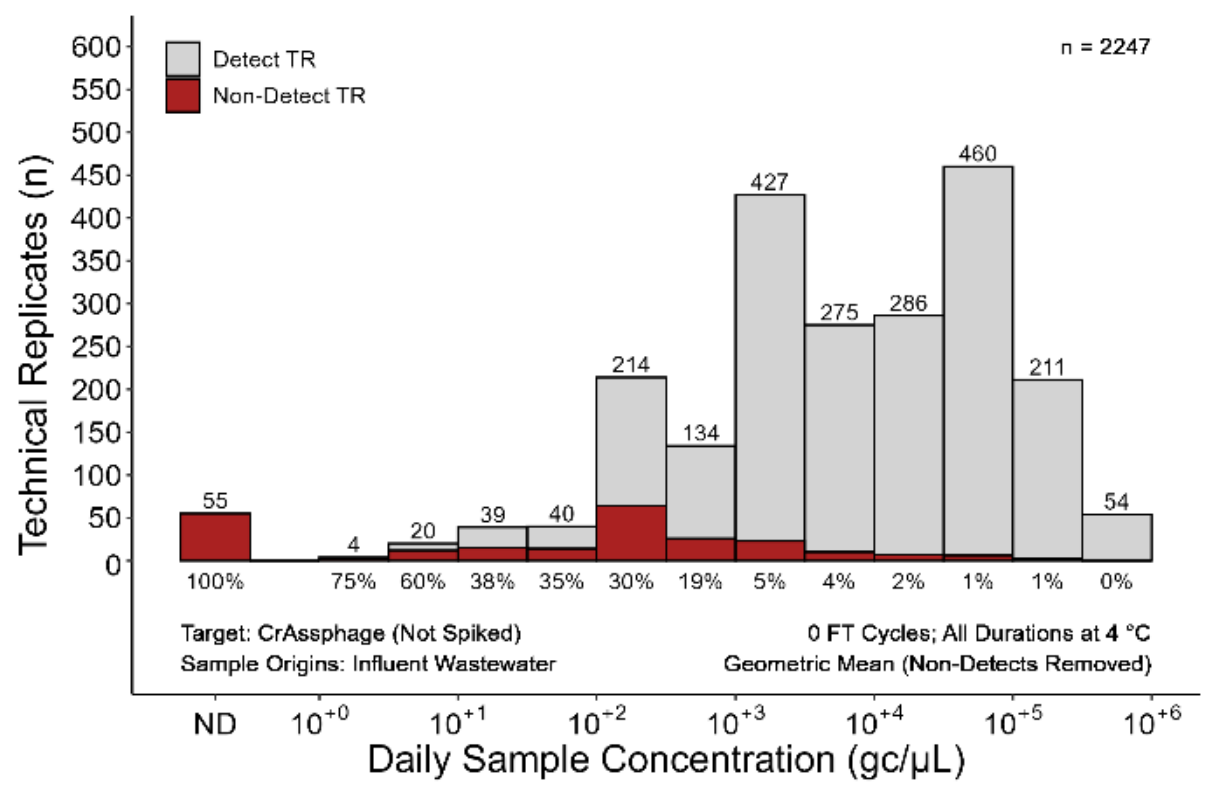

a) Continuous Methodological Variability for CrAssphage is visualised as a scatter plot of DS:TR ratio against DS concentration.

b) Continuous Methodological Variability for CrAssphage is visualised as a histogram of DS:TR ratio.

c) Discrete Methodological Variability for CrAssphage is visualised as a bar chart of detect/non-detect TRs against DS concentration range.

Fig. S12(a-c) Visualisation of Methodological Variability (MV) for the CrAssphage gene target.

Total Estimated SARS-CoV-2 Composite Metric

a)

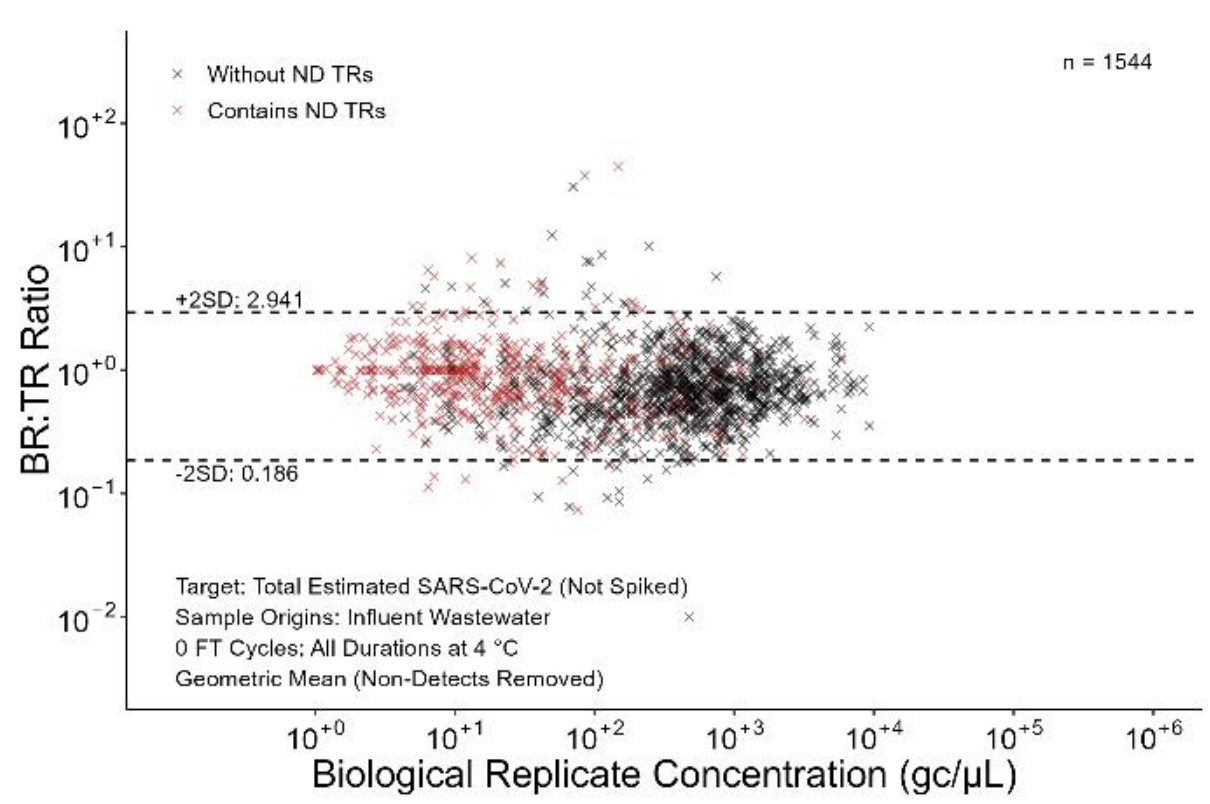

b)

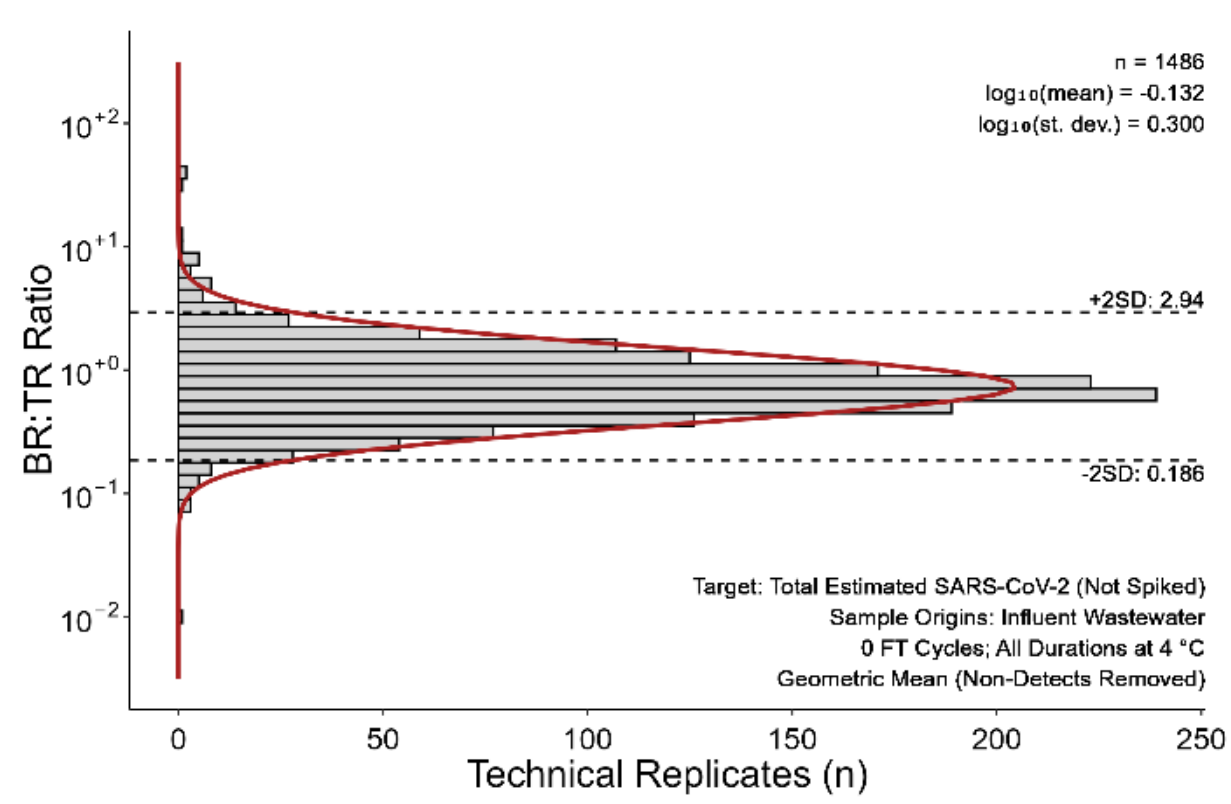

c)

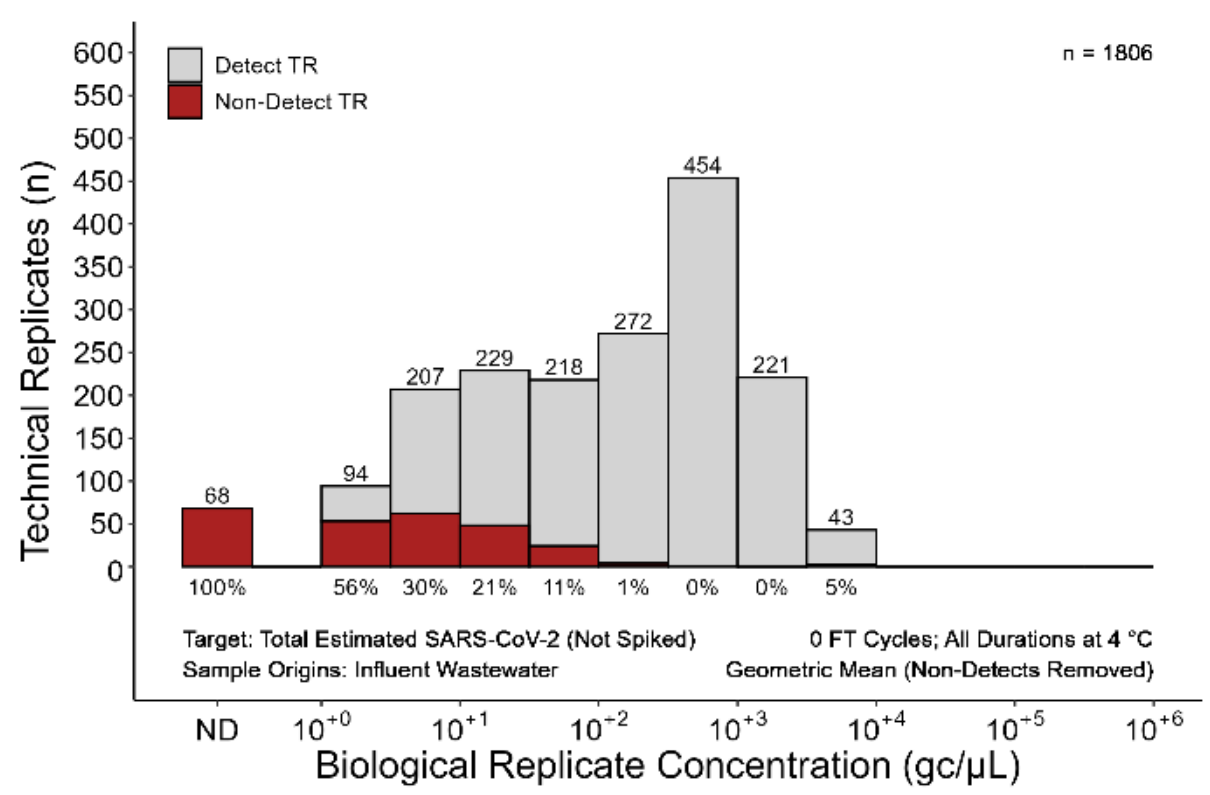

a) Continuous Analytical Variability for Total Estimated SARS-CoV-2 is visualised as a scatter plot of BR:TR ratio against BR concentration.

b) Continuous Analytical Variability for Total Estimated SARS-CoV-2 is visualised as a histogram of BR:TR ratio.

c) Discrete Analytical Variability for Total Estimated SARS-CoV-2 is visualised as a bar chart of detect/non-detect TRs against BR concentration range.

Fig. S13(a-c) Visualisation of Analytical Variability (AV) for Total Estimated SARS-CoV-2 composite metric.

a)

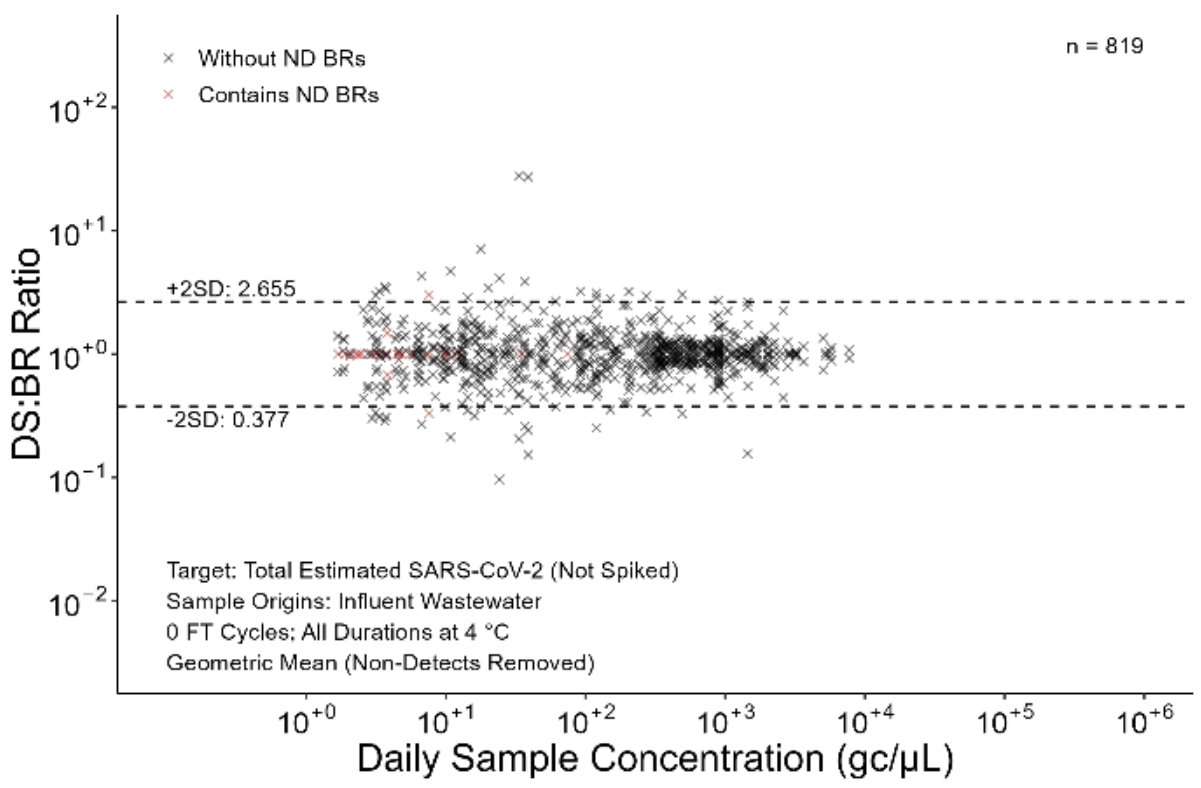

b)

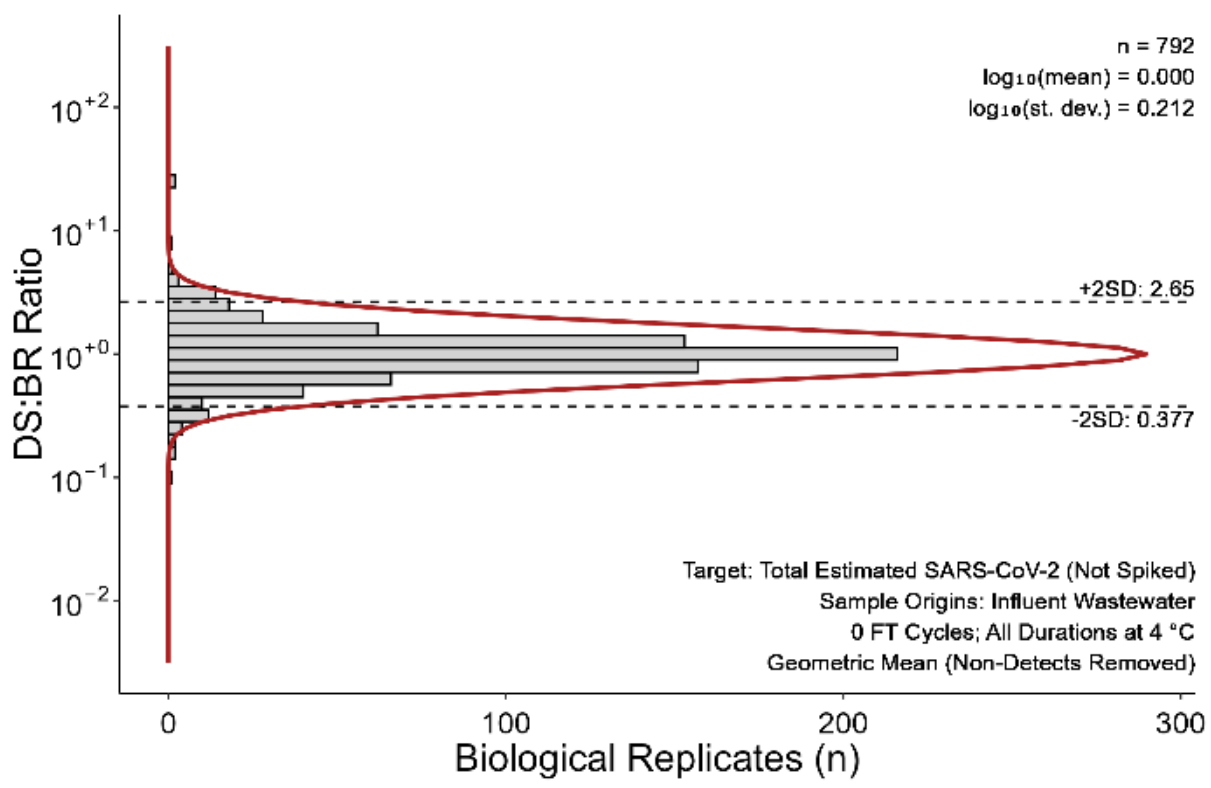

c)

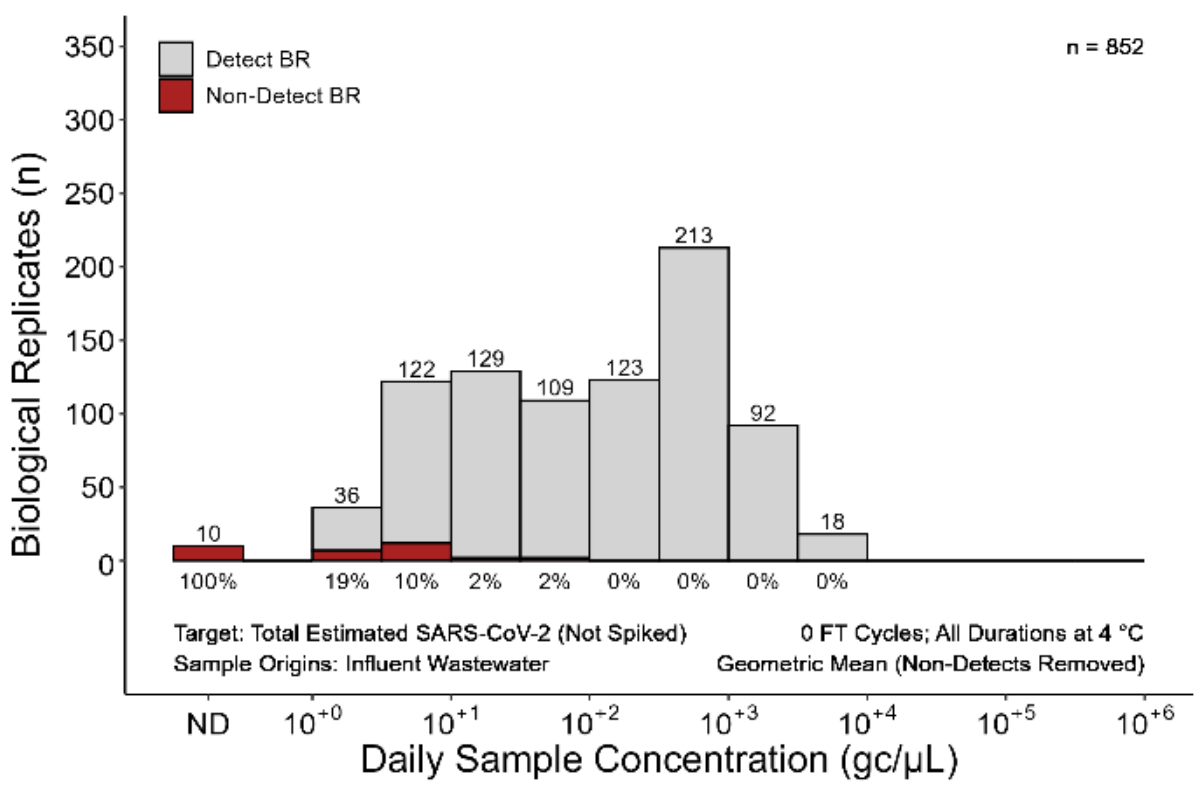

a) Continuous Processing Variability for Total Estimated SARS-CoV-2 is visualised as a scatter plot of DS:BR ratio against DS concentration.

b) Continuous Processing Variability for Total Estimated SARS-CoV-2 is visualised as a histogram of DS:BR ratio.

c) Discrete Processing Variability for Total Estimated SARS-CoV-2 is visualised as a bar chart of detect/non-detect BRs against DS concentration range.

Fig. S14(a-c) Visualisation of Processing Variability (PV) for Total Estimated SARS-CoV-2 composite metric.

a)

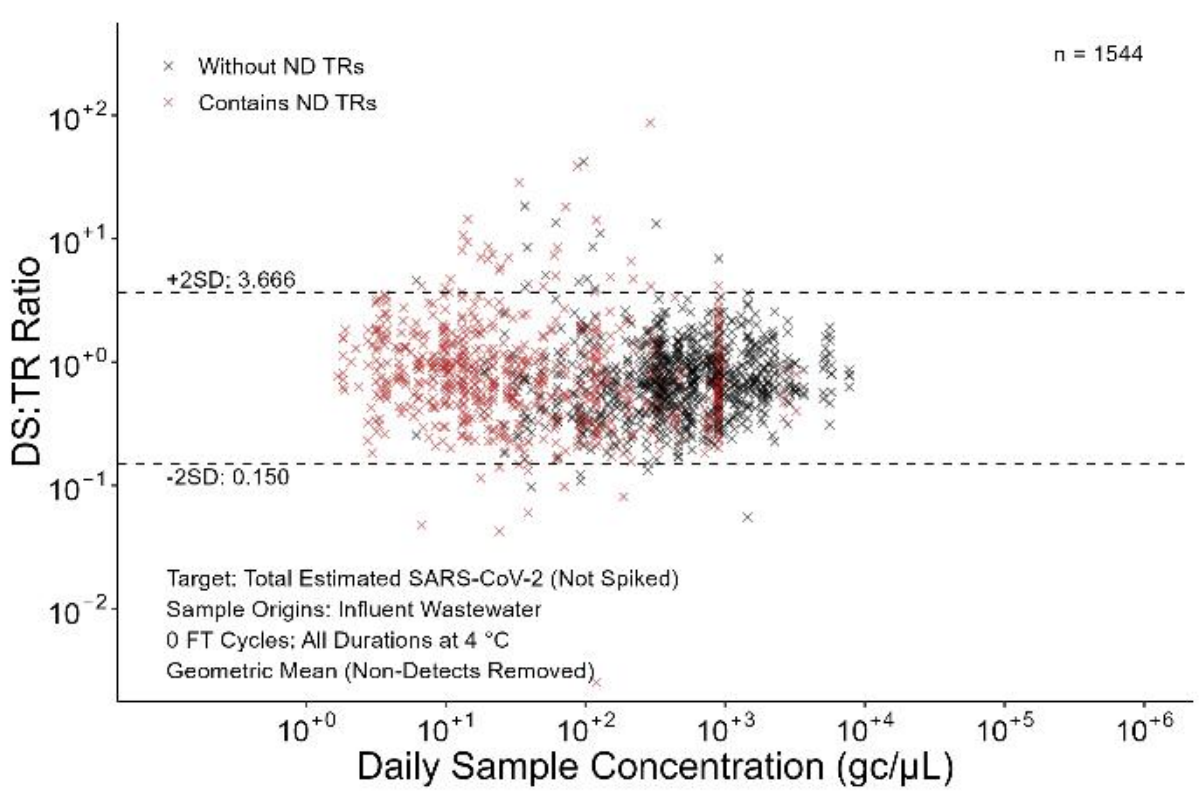

b)

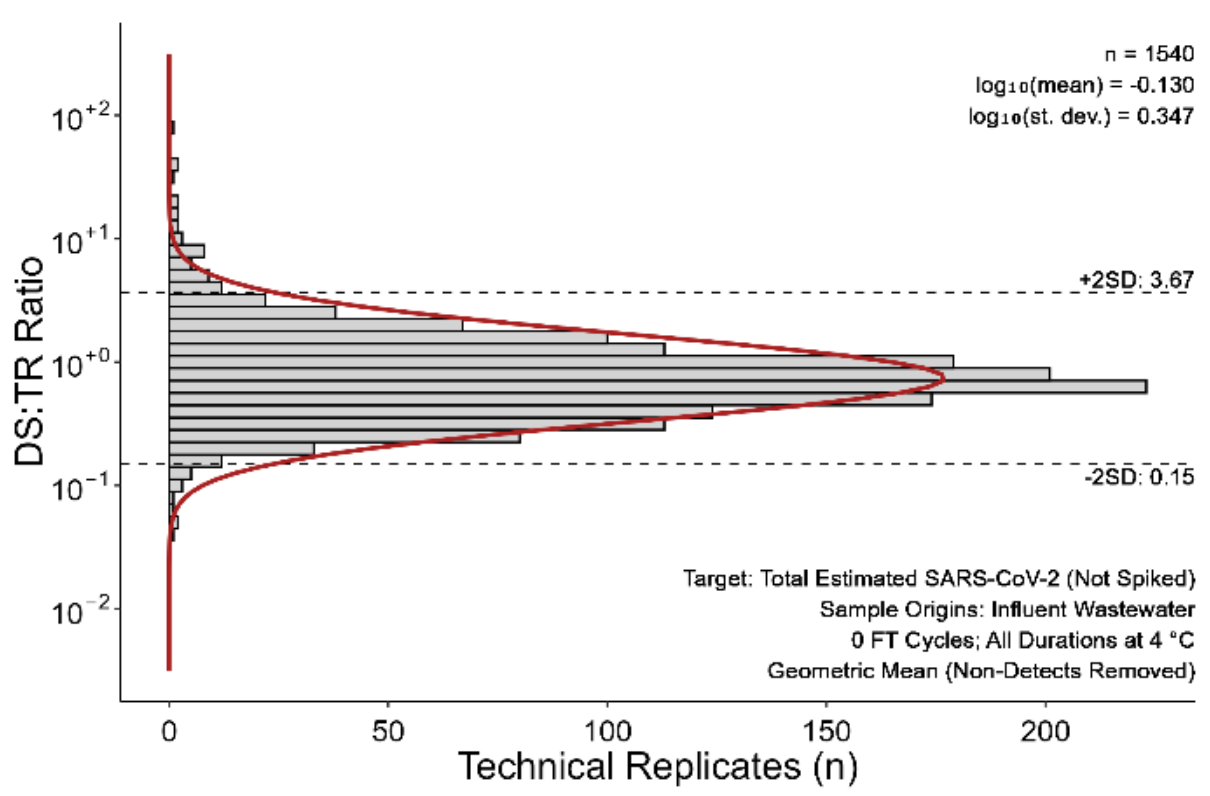

c)

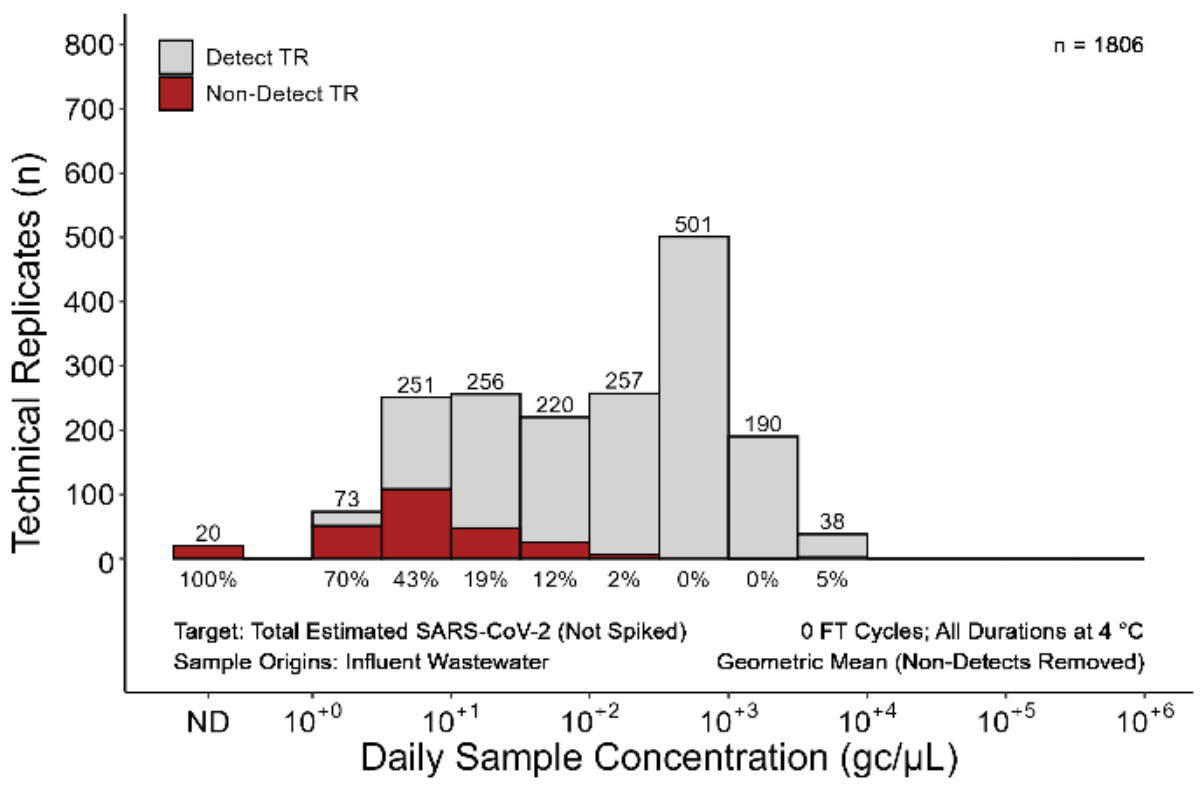

a) Continuous Methodological Variability for Total Estimated SARS-CoV-2 is visualised as a scatter plot of DS:TR ratio against DS concentration.

b) Continuous Methodological Variability for Total Estimated SARS-CoV-2 is visualised as a histogram of DS:TR ratio.

c) Discrete Methodological Variability for Total Estimated SARS-CoV-2 is visualised as a bar chart of detect/non-detect TRs against DS concentration range.

Fig. S15(a-c) Visualisation of Methodological Variability (MV) for Total Estimated SARS-CoV-2 composite metric.

a)

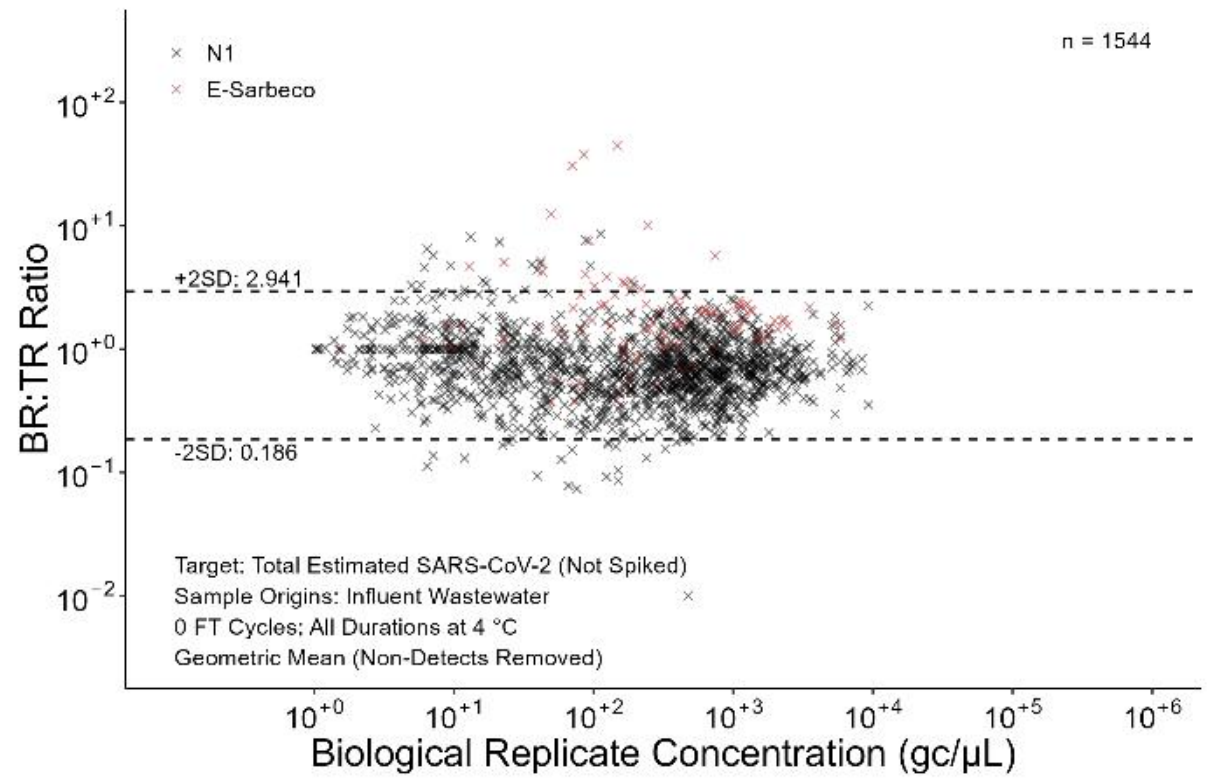

b)

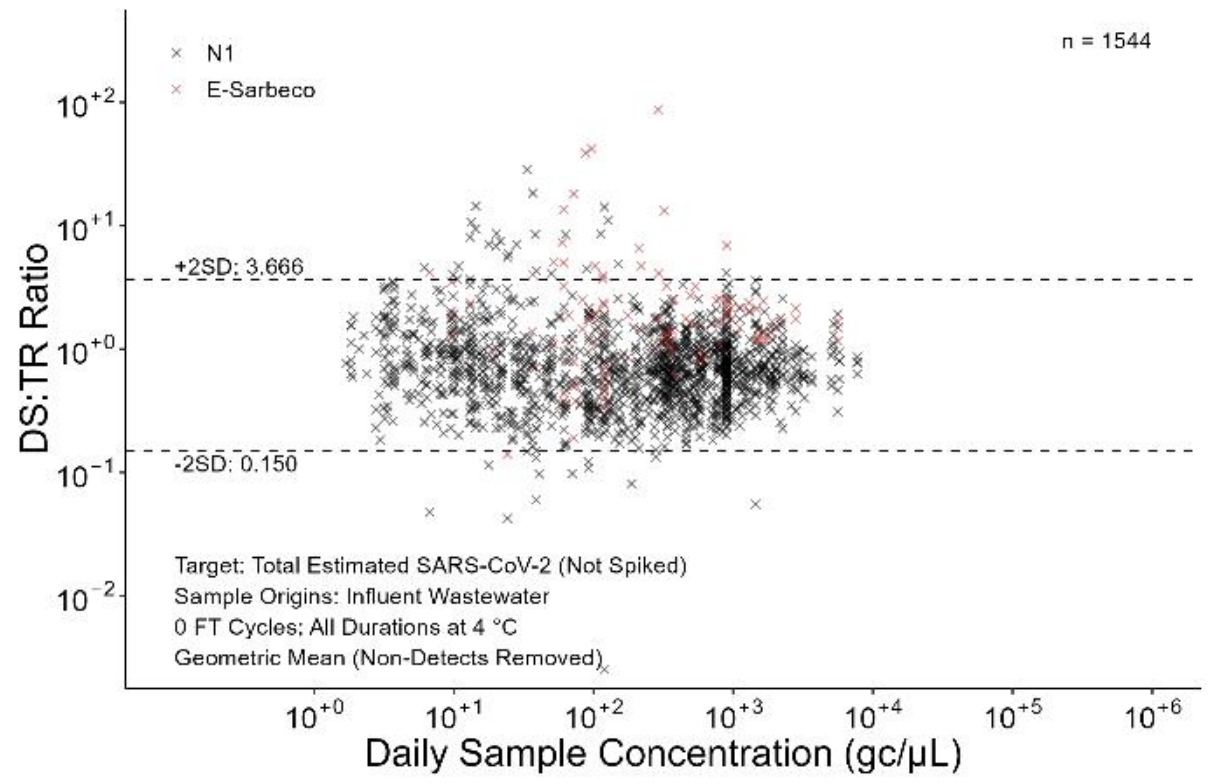

Fig. S16(a-b) Visualisation of (a) Processing Variability (PV) and (b) Methodological Variability (MV) for Total Estimated SARS-CoV-2 composite metric, identifying TR data originating from SARS-CoV-2 E-Sarbeco and N1 gene targets.

a)

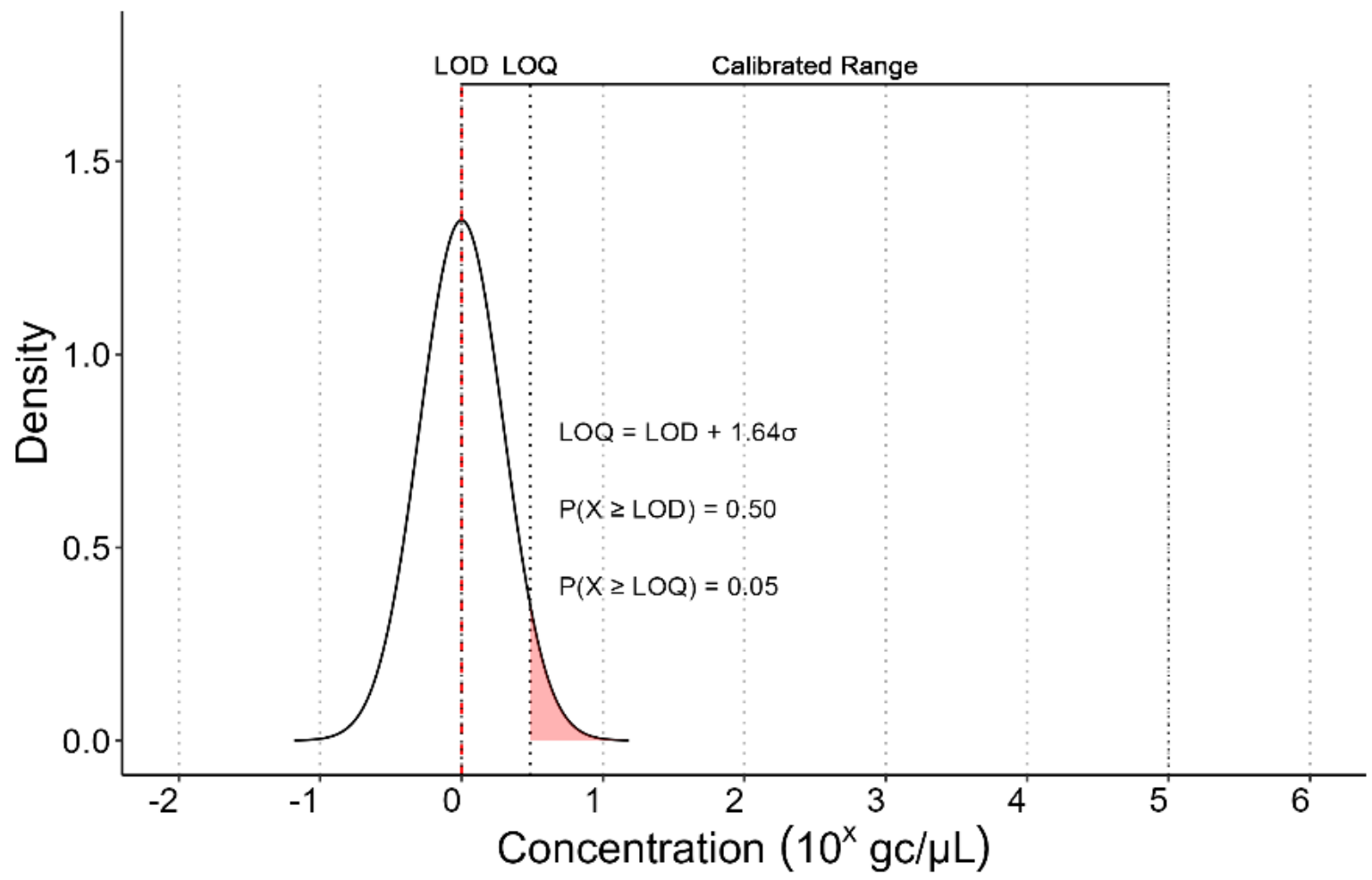

b)

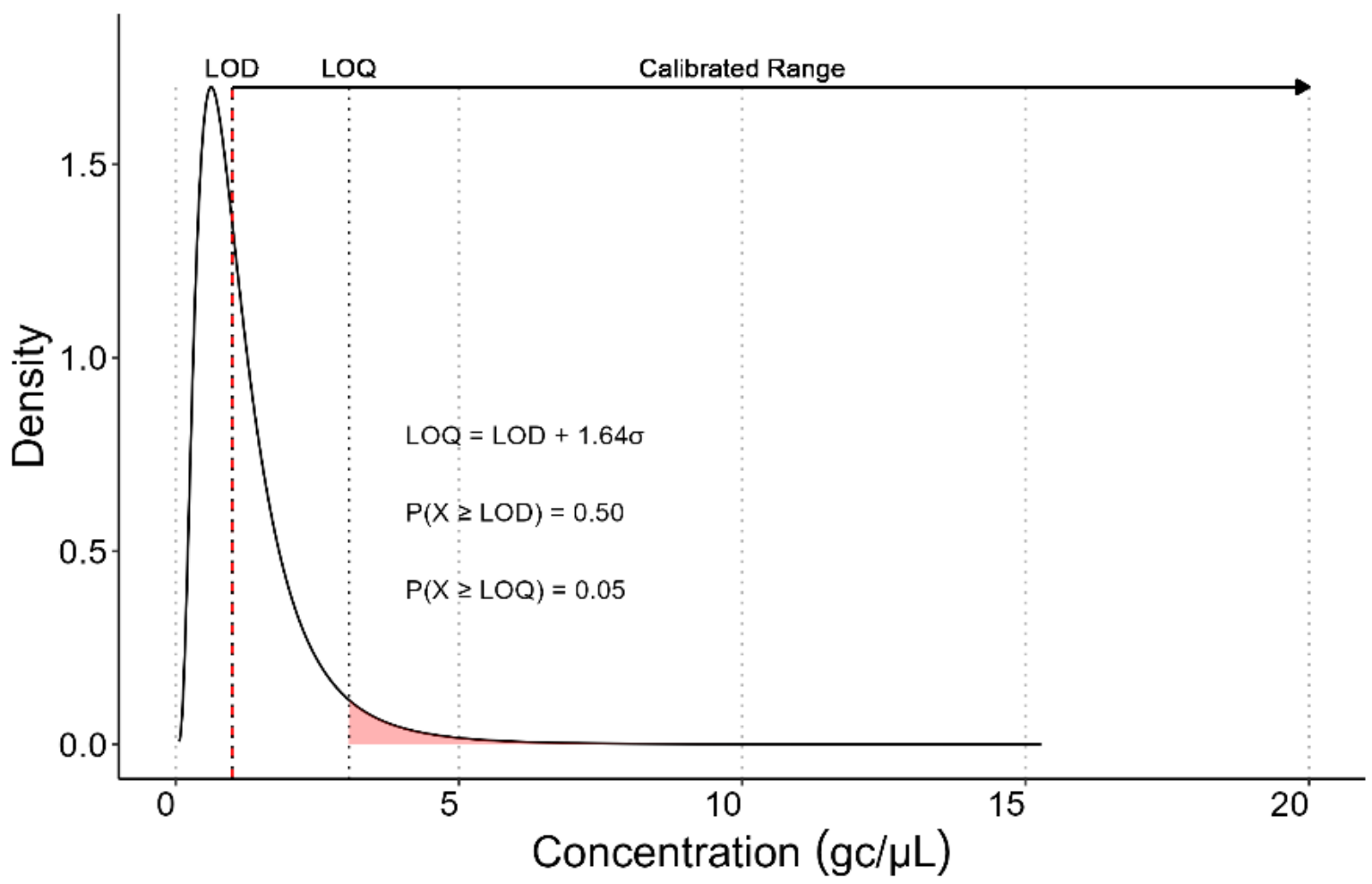

Fig. S17(a-b) Determination of LOQ from the LOD using (a) additive model for the determination of LOQ, applied to log-transformed concentration data, and (b) additive model visualised on the linear scale. The LOQ is the result of a mathematically analogous transformation, whereby the LOD was multiplied by the 95th percentile of the Methodological Variability ($MV_{95\%}$).
